# Cell-type-specific regulators landscape and regulatory mechanisms underlying pyroptosis in uterine corpus endometrial carcinoma

**DOI:** 10.7150/jca.100547

**Published:** 2025-01-01

**Authors:** Hongrui Guo, Wufeng Qin, Jinpeng Li, Fucheng Wang, Jiaolin Yang, Yaling Wang, Xinglin Zhang, Yuanyuan Ding, Kaiwen Ting, Xia Li, Jingru Ji, Yanyan Han, Ailing Hui, Huancheng Su, Sanyuan Zhang, Zhe Wang

**Affiliations:** 1 Shanxi Medical University, Taiyuan 030001, China.; 2 Department of Gynecology, First Hospital of Shanxi Medical University, Taiyuan 030001, China.; 3 Department of Obstetrics and Gynecology, Taiyuan People's Hospital, Taiyuan 030009, China.; 4 College of nursing, Shanxi medical university, Taiyuan 030001, China.; 5 Department of Obstetrics and Gynecology, First Hospital of Shanxi Medical University, Taiyuan 030001, China.; 6 Department of Gynecology, Jiaocheng County People's Hospital, No. 25 Tianning Street, Jiaocheng County, Lüliang City, Shanxi Province, China.; 7 Department of Ultrasound, Jiaocheng County People's Hospital, No. 25 Tianning Street, Jiaocheng County, Lüliang City, Shanxi Province, China.

**Keywords:** Pyroptosis, Endometrial cancer, Single-cell sequencing, Ligand-receptor, TNF pathway.

## Abstract

**Background**: Endometrial cancer (UCEC) has a significant detrimental effect on patient quality of life. Although pyroptosis-related genes have been reported to contribute to tumor pathogenesis, the specific mechanism of pyroptosis in patients with UCEC remains elusive.

**Methods:** We provide an overview of the landscape of pyroptosis-related genes in UCEC tissues through single-cell RNA sequencing (scRNA-Seq) datasets from the tissues of UCEC of 6089 cells. In addition, pyroptosis-related gene expression pattern was verified based on the RNA-Seq datasets, and observation of abnormal pathological characteristics of UCEC tissue.

**Results:** The pyroptosis-related gene *IL-6* is specifically upregulated in epithelial cells and dysregulates cell population proliferation and enhances apoptosis. The upregulation of *BAX* and *TNF* expression in macrophages induces infiltration of aberrantly activated macrophages, which display dysfunctional differentiation in tumor tissues, altered immune responses, and activation of the tumor necrosis factor (TNF) pathway in UCEC macrophages. In addition, dysregulation of pyroptosis-related genes induces aberrant cell-cell communication in tumor tissues and mediates ligand-receptor interactions between various cell types in UCEC via the *TNF* signaling pathway to promote cancer progression. Quantitative real-time (PCR) and immunohistochemistry were used for the *in vitro* experimental validation.

**Conclusion:** Pyroptosis-related genes can serve as biomarkers for UCEC, playing a role in early disease diagnosis, helping to predict patient prognosis, and guiding the selection of personalized treatment options.

## Introduction

Endometrial cancer (UCEC), a common malignancy of the female reproductive system, poses significant health risks and has a detrimental impact on the quality of life of affected women 1. UCEC originates in the epithelium of endometrial glands and is the sixth most commonly diagnosed cancer in women. In 2020, there were 417,000 new cases and 97,000 deaths worldwide 2. With increasing rates of obesity and other factors, the incidence of UCEC has been increasing annually, placing enormous pressure on public health systems and increasing the burden on medical resources 3. Therefore, early detection and intervention of UCEC should receive greater attention 4.

Programmed cell death, one of the most extensively studied cell death pathways in recent years, includes mechanisms such as apoptosis 5, 6, autophagy, pyroptosis 6, 7, ferroptosis 8, PANoptosis, necroptosis 6, and cuproptosis 9, which act as powerful triggers for inflammatory responses, particularly necroptosis and pyroptosis 10. Pyroptosis, a recently recognized form of programmed cell death 5, differs from apoptosis in its inflammatory nature and involvement of specific gasdermin proteins 11, 12. Conversely, pyroptosis is triggered by the activation of inflammatory caspases (such as caspase-1) and cleavage and activation of gasdermin family proteins, leading to rupture of the cell membrane and formation of "pyroptotic bodies" containing *IL1B* and IL18, which induce an inflammatory response around cells 7. Pyroptosis is characterized by cell swelling, plasma membrane rupture, and release of pro-inflammatory cytokines 13, 14, 15 that regulate the tumor microenvironment, promote the formation and maintenance of cancer stem cells, and are closely related to the growth, metastasis, and recurrence of cancer cells after treatment 16. Furthermore, when the tumor microenvironment is accompanied by an inflammatory response, various cytokines and chemokines, such as tumor necrosis factor (TNF) α, interferon (IFN) γ, and chemokine (C-X-C motif) ligand (CXCL) 9, 10, 11. However, a prolonged inflammatory environment can also lead to exhaustion of immune cells, thereby reducing their anti-tumor activity 17. Pyroptosis can recruit immune cells and enhance the proliferation and survival of cancer cells, potentially creating a positive feedback loop that exacerbates the disease state 18.

The goal is to find gene markers that can better evaluate the clinical significance and biological mechanisms of UCEC, as well as improve predictions regarding the effectiveness of immunotherapy and patient outcomes. We examined the mechanisms of pyroptosis and its impact on the tumor microenvironment at both pathological and molecular levels using single-cell transcriptome and sequencing datasets. We explored the cell type-specific regulation of pyroptosis-related genes and the distinct functions exerted in the progression of UCEC at the single-cell level. We also determined whether genes related to pyroptosis play a role in dysregulated pathways affected by dysfunctional biological relationships. In summary, our study found that pyroptosis-related genes may serve as potential biomarkers for the diagnosis and prognosis of UCEC, which could help improve diagnostic accuracy and provide a scientific direction for developing more effective treatment strategies.

## Method

### Single-cell RNA sequencing (scRNA-seq) dataset processing and analysis

Raw reads from human endometrial cell fastq files were processed using the Cell Ranger Software Suite (10× Genomics Cell Ranger 4.0.0), with refdata-gex-GRCh38-2020-A as the reference for mapping reads to the human genome (GRCh38/hg38). This processing generated unique molecular identifier (UMI) matrices. The outputs from Cell Ranger were then imported into Seurat using the 'Read10X' function. Quality control measures included removing cells with UMI counts in the upper 10% for each sample to eliminate potential outliers. Additionally, cells with fewer than 500 UMIs detected or those with more than 40% mitochondrial UMIs were filtered out. Genes expressed in fewer than 1 cell were also removed. Quality control was managed using the 'Seurat' package (version 4.0). For normalization, a global scaling method called LogNormalize was applied to normalize gene expression measurements across cells, considering both characteristic and total expression levels. Differentially expressed genes (DEGs) were identified using the FindMarkers function embedded in Seurat to precisely annotate cell types; we manually curated genetic markers for each cell type. Most markers that distinguished between different cell types were retrieved from the Cell Marker Database (https://www.labome.com/method/Cell-Markers.html), with the following cutoff thresholds: Benjamin-Hochberg's adjusted p value < 0.01 and Fold Change > 1.5. The DEGs were loaded into the cluster profile package for Gene Ontology (GO) enrichment analyses. Pathways with an adjusted p value of < 0.05 were considered significantly enriched. We then performed a gene set enrichment analysis (GSEA) using the gsea function embedded in the cluster profiler package to detect which gene set was significantly enriched in each specific cell cluster.

### Dataset source and preprocessing

Publicly available gene expression data and complete clinical annotations were obtained from TCGA and ICGC. We selected patients with survival information for further evaluation and excluded others. A total of 401 eligible tumor samples from the TCGA-UCEC cohort were included for subsequent analysis. All samples were used for mutation analysis. Samples from the TCGA-UCEC cohort were utilized for unsupervised clustering and consensus clustering analyses. RNA sequencing data, represented as (Fragments Per Kilobase of exon model per Million mapped fragments) FPKM values of gene expression, were downloaded from the Genomic Data Commons (GDC, https://portal.gdc.cancer.gov/) using R packages TCGA and ICGC Bioconductor. These packages were specifically developed for integrative analyses of GDC data 19. Additionally, somatic mutation data were acquired from the TCGA database. Data for CNV analysis were downloaded from the TCGA-UCEC dataset. The data were analyzed using R (version 4.2.3) and R Bioconductor software packages.

### Unsupervised clustering and consensus clustering analysis

We extracted total of 51 pyroptosis-related genes from the TCGA and ICGC datasets to identify distinct modification patterns mediated by these genes. The analyzed genes included *BAX, CHMP2A, CHMP2B, CHMP4A, CHMP4B, IL1A, IL1B, IL-6, ect.* Unsupervised clustering analysis was applied to identify distinct patterns of pyroptosis-related genes based on their expression. This analysis helped classify patients for further analysis. The number of clusters and their stability were determined using a consensus clustering algorithm 20. These analyses were performed using the ConsensusClusterPlus package with 1000 iterations to ensure the stability of the classification 21.

### Enrichment gene set variation analysis with functional annotation

We conducted GSVA enrichment analysis using the “GSVA” R package to explore differences in biological processes among pyroptosis-related genes. GSVA is a non-parametric and unsupervised method widely used to estimate variation in pathway activity and biological processes across samples in an expression dataset. Statistical significance was determined with an adjusted P-value of less than 0.05. The clusterProfiler R package was used to perform functional annotation for pyroptosis-related genes 22, with the cutoff value of FDR set at less than 0.05.

### TME cell infiltration estimation

To quantify the relative abundance of each cell type infiltrating the UCEC TME, we employed the single-sample gene-set enrichment analysis (ssGSEA) algorithm. The gene set used to mark each type of TME infiltrating immune cell was obtained from the study by Charoentong, which includes various human immune cell subtypes including activated dendritic cells, macrophages, natural killer T cells, and regulatory T cells, among others 23, 24. The ssGSEA-derived enrichment scores were used to represent the relative abundance of each TME infiltrating cell in each sample. Additionally, we calculated tumor purity, stromal, immune, and ESTIMATE scores for each sample using the ESTIMATE algorithm. This algorithm assesses the tumor component in each sample 25. Subgroup comparisons were analyzed using the “limma” R package, allowing for a detailed examination of score variations.

### Identification of DEGs between distinct pyroptosis-related genes

To explore the diversity of pyroptosis-related genes, we divided patients into three groups based on the expression of pyroptosis-related genes. We used the empirical Bayes method provided by the "limma" R package to identify DEGs between the distinct patterns. The criterion for determining significant DEGs was set at an adjusted P-value threshold of less than 0.001. Further analysis of pathways associated with these DEGs was conducted using the GO and KEGG databases.

### Correlation between the risk score and other related biological processes or clinical features

We performed a correlation analysis to further reveal the association between the pyroptosis-related gene signature and some related clinical features, including (1) immune-checkpoints; (2) TMB; (3) TIDE; (4) age; (5) tumor grade; (6) several drugs including vinblastine, lenalidomide, temsirolimus, bexarotene; (7) ESTIMATES score; (8) immune score; (9) stromal score; (10) tumor purity.

### Weighted gene co-expression network analysis and prognostic model construction

WGCNA is a method for studying the correlation between biological networks and diseases. We used the R package WGCNA 16 for WGCNA, with the aim of using immune-related genes in the TCGA-UCEC dataset to identify key modules related to myeloid cells, lymphocytes, endothelial cells, fibroblasts, smooth muscle cells, and epithelial cells. To determine genes associated with prognosis, we employed random forest for binary variable and lasso regression. The predictive value of the constructed prognostic model was evaluated using the ROC curve.

### Tissue selection

We collected endometrial tissues from 21 patients with endometrial tumors at the First Hospital of Shanxi Medical University and 21 patients with normal endometrial conditions. All patients underwent hysterectomy (Table 1, Tables S1-6). The selection of tissues was based on histopathological analysis conducted by a pathologist with more than two years of clinical experience. Each tissue sample was 1.0 centimeters long, 0.5 centimeters wide, and 0.5 centimeters thick. Adjacent tissues were excised 1 centimeter from the edge of the tumor. RT-qPCR detection was performed on six tissues. Immunohistochemistry was conducted on 36 paraffin-embedded tissue specimens. One tumor tissue and one normal tissue were selected from the collected samples for single-cell RNA sequencing (scRNA-seq).

### Immunohistochemistry

Proportion of antibody dilution: *BAX* (1: 200) (ab32503, Abcam lnc, Cambridge, MA, USA), CHMP2A (1: 200) (ab67064, Abcam lnc, Cambridge, MA, USA), CHMPB (1: 200) (ab157208, Abcam lnc, Cambridge, MA, USA), *IL-1A* (1: 200) (ab300499, Abcam lnc, Cambridge, MA, USA), *IL-1B* (1: 200) (ab283818, Abcam lnc, Cambridge, MA, USA), VPS24 (1: 200) (ab175930, Abcam lnc, Cambridge, MA, USA). Tissue sections, 4 µm thick, were deparaffinized in xylene and rehydrated through a graded series of ethanol. Endogenous peroxidase activity was blocked using 3% hydrogen peroxide. Sections were incubated with the primary antibody at room temperature for 4 hours, followed by incubation with the appropriate secondary antibody. The tissue sections were then stained with 3,3'-diaminobenzidine and counterstained with hematoxylin. Positive rates of IHC were analyzed using ImageJ software.

### RT-qPCR

Total RNA was extracted from tissue samples using Trizol reagent (Ambion, USA). The mRNA was then reverse transcribed into cDNA using the cDNA Synthesis SuperMix (TransGen Biotech, Beijing). PCR amplification was conducted using a PCR machine (Bioer Technology, GeneMax Tc-s-B, China) using the following cycling parameters: an initial denaturation at 95 ℃ for 5 min, followed by 40 cycles of denaturation at 95 ℃ for 10 s, and annealing at 60 ℃ for 30 s. Gene expression levels were quantified using the ΔΔCt method. The experiments were performed in triplicates to ensure reproducibility. The Primer nucleotide sequence was showed in Table.S7.

### Construction of the Risk Model

Least absolute shrinkage and selection operator (LASSO) Cox regression was performed using “glmnet” in the R package. Application of the subsequent formula was used to count the risk score: Risk score = coef (gene1) × expr (gene1) + coef (gene2) × expr (gene2) + …… + coef (gene n) × expr (gene n) in which coefifi corresponded to the coefficients, coef (gene n) corresponds to the coefficient of pyroptosis-related genes correlated with survival, and expr (gene n) corresponded to the expression of pyroptosis-related genes. Genes showing significant prognostic impact were selected for further analysis. We then applied PCA to construct the HOX-relevant gene signature.

### Statistical analysis

Spearman and distance correlation analyses were used to assess the correlation between the expression of TME-infiltrating immune cells and the regulators of pyroptosis-related gene expression. The "surf" R package was used to establish a cutoff point based on the correlation between the risk score and patient survival. The Kaplan-Meier method was used to generate survival curves for prognostic analysis. The significance of these differences was determined by the log-rank test. All statistical tests were two-sided, with a P-value of less than 0.05 considered significant. All data processing was performed using R software version 4.2.3.

## Results

### Landscape of pyroptosis-related genes expression revealed by scRNA-seq analysis among six cell types in UCEC

**Figure 1:**
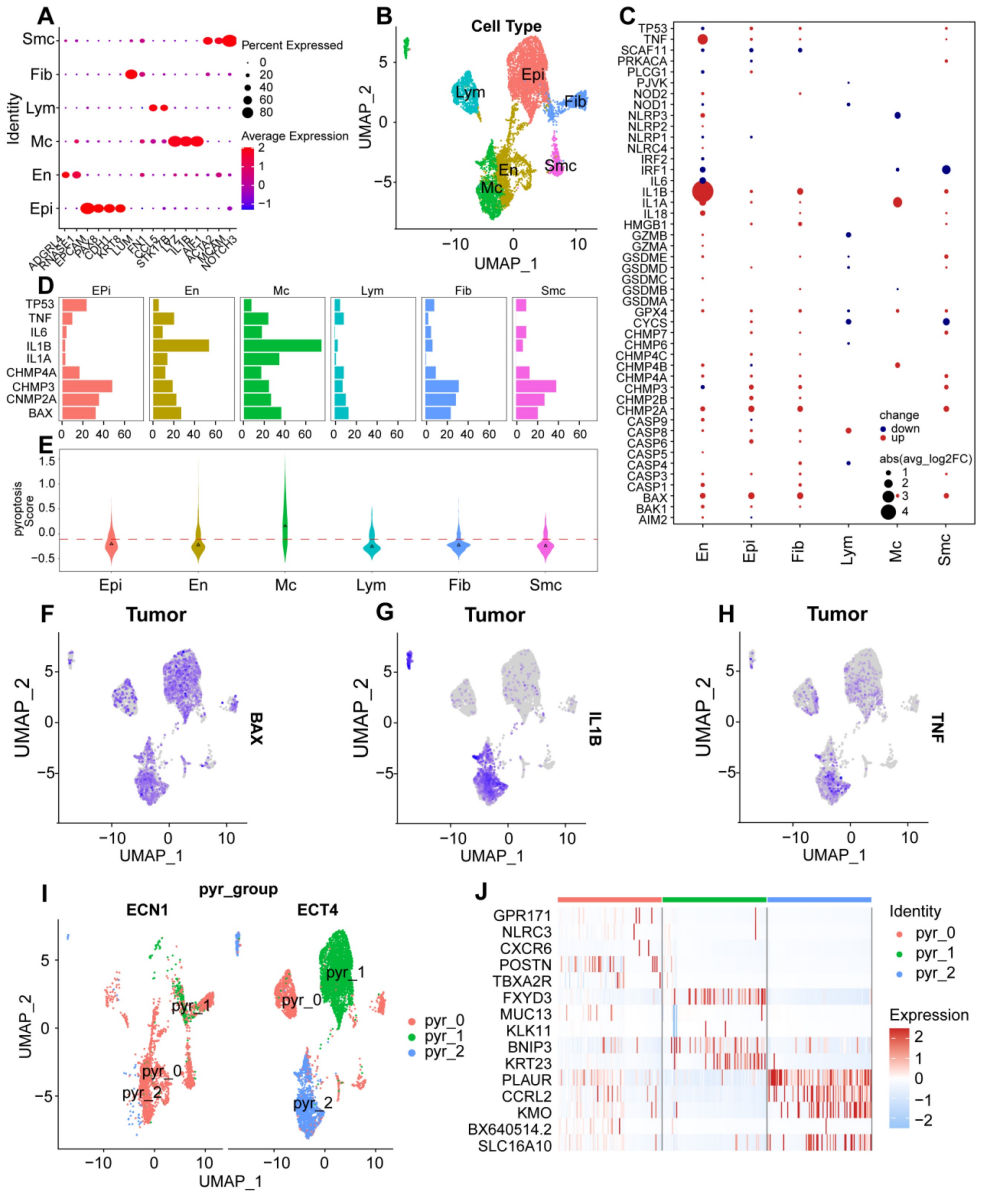
** Expression profile of pyroptosis regulators in UCEC cell types through single-cell RNA-seq (scRNA-seq). (A)** The plot shows identified cell types and annotated using classical marker genes. **(B)** UMAP graph showing the expression of six cell types, including epithelial cells, endothelial cells, macrophages, lymphocytes, fibroblasts, and smooth muscle cells, between UCEC patients and healthy individuals. **(C)** Differences in the expression of pyroptosis regulators in six cell types. The size of dots represents the percentage of cells expressing the gene. The color of the dots represents the average expression level; red indicates high expression, and blue indicates low expression. **(D)** Percentage of pyroptosis regulators in each cell type. **(E)** Pyroptosis scores for each cell type. **(F-H)** Mean expression levels of *BAX, IL1B* and *TNF* in tumor tissues. **(I)** UMAP for dimensionality reduction and clusters identified by pyroptosis regulators showing three clusters. **(J)** Heatmap of DEGs of unsupervised clustering by three clusters of pyroptosis regulators.

To characterize various cell types in UCEC, we applied scRNA-seq data for normal endometrial tissue and endometrial tumor tissue, analyzed scRNA-seq datasets comprising 9012 cells from patients with UCEC and healthy individuals, and constructed a uniform manifold approximation and projection (UMAP) visualizing the results of cell clustering (Fig. S1A). Of these cells, 6089 were obtained from tumor samples and 2923 from healthy tissues, including six cell types (myeloid cells, lymphocytes, endothelial cells, fibroblasts, smooth muscle cells, and epithelial cells), which were annotated using classical marker genes (Figs. 1A-B and S1B).

Violin plots were used to illustrate the expression distribution of marker genes in various cell types (Fig. S1C). To further explore the relationship between pyroptosis-related genes and UCEC, we performed expression and proportion analyses of pyroptosis-related genes in different cell types. RNA expression of the main pyroptosis-related genes, including *TP53, TNF, IL1B, IL1A, BAX, CHMP4A, CHMP3, and CHMP2A*, was significantly higher in UCEC cells than in the normal group (Fig. 1C). In particular, the UCEC group showed high expression of *TP53, CHMP2A, CHMP3, CHMP4A*, and* BAX* in epithelial cells, whereas *TNF, IL1A, IL1B, IL-6, CHMP4A*, and *BAX* were highly expressed in macrophages (Fig. S1D). To better understand the proportion of pyroptosis-related genes, we explored the percentage expression of these genes in each cell type and found that the proportion of expression quantity of *IL1B, CHMP2A, CHMP3, CHMP4A*, and *BAX* in various UCEC cell types was relatively high (Fig. 1D). Furthermore, to identify the global expression of pyroptosis-related genes, we used pyroptosis-related genes as a score to calculate the overall gene expression patterns using the Add Module Score method, which revealed that the highest score was found for macrophages, whereas epithelial cells had the lowest score (Fig. S1E). Compared with the mean score for the six cell types, the macrophage score was above the mean value (Fig. 1E). Subsequently, we analyzed the expression of genes related to pyroptosis in normal and tumor tissues. The uniform manifold approximation and projection (UMAP) diagram revealed that *BAX, IL1B,* and *TNF* were predominantly expressed in epithelial cells and macrophages in tumor tissues (Figs. 1F-H and S1F-H). These results indicate that pyroptosis-related genes are expressed in UCEC and play a crucial role in tumor regulation through abnormally high expression.

**Figure 2:**
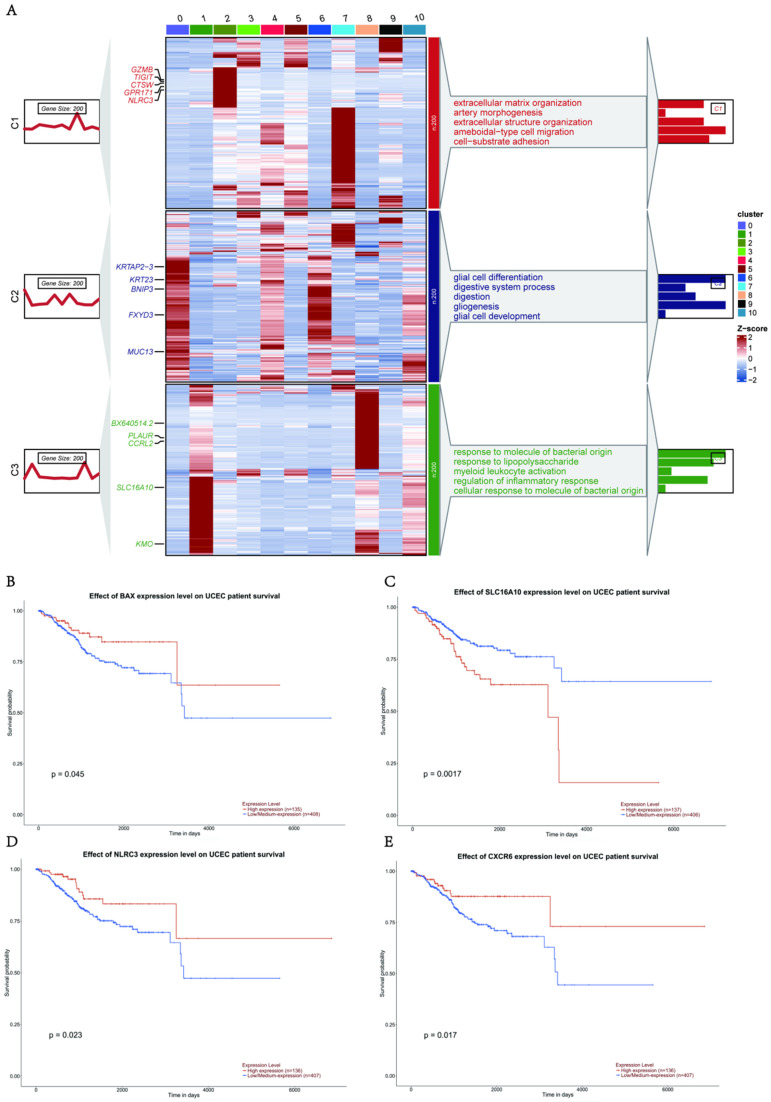
** Expression patterns of pyroptosis regulators in patients with UCEC and healthy individuals. (A)** Expression patterns of pyroptosis-regulator clusters in patients with UCEC and healthy individuals. Heatmap of DEGs of unsupervised clustering from UCEC and healthy groups; the DEGs in each module were annotated; the line graph shows the trend in gene module expression, and the text on the right shows the enriched pathways for each module gene. **(B-E)** Kaplan-Meier curves showing progression-free survival in* GEPIA 2* in the UCEC Cohort stratified according to high vs. low expression of *BAX* (B), *SLC16A10* (C), *NLRC3* (D), and* CXCR6* (E).

Given the enhanced expression of the pyroptosis-related genes described above, we explored the expression and regulation of these genes in different cell types. Heterogeneity of the cell-type distribution was applied for dimensionality reduction using pyroptosis-related genes and clusters were visualized using UMAP. We detected three heterogeneous cell compositions among these cells, and found that pyr-0 was mainly clustered in lymphocytes, pyr-1 in epithelial cells, and pyr-2 in macrophages (Fig. 1I). To determine the cellular identity of each cluster, we generated cluster-specific marker genes via differential gene expression analysis using well-known cell type markers for epithelial cells in pyr-1, such as *KRT23*, to determine the regulation of the clusters. In addition, we identified genes in pyr-2, such as *PLAUR* and *CCRL2*, as macrophage-related genes (Fig. 1J). To explore the characteristics of pyroptosis-related genes and their different biological roles, unsupervised cluster analysis using pyroptosis-related genes revealed three patterns of expression (clusters 1, 2, and 3). Next, we investigated the molecular regulation of the pyroptosis-related genes in UCEC in the three clusters (Fig. 2A).

In cluster 1 (pyr-0), genes, including *GPR171* and *NLRC3*, regulate pathways related to tumor occurrence and development, such as cell-substrate adhesion. In group 2 (pyr-1), pathways such as glial cell differentiation, development, and gliogenesis are regulated by *FXYD3*, *MUC13*,* BNIP3*, and *KRT23*. In cluster 3 (pyr-2), *BX640514.2*, *PLAUR*, *CCRL2*, *SLC16A10,* and* KMO* regulate myeloid leukocyte activation and inflammatory response pathways. Overall survival analysis showed that the differences in the expression of these genes had different effects on patient prognosis, revealing the clinical value of DEGs in pyroptosis-related clusters in guiding patient prognosis. *BAX*,* KMO,* and* SLC16A10* are expressed in macrophages. High expression of *BAX* and *KMO* and low/medium expression of *SLC16A10* were associated with better survival status (Figs. 2B-C). *NLRC3 and CXCR6* are expressed in lymphocytes. Patients with high expression of these genes showed better survival status (Figs. 2D-E). These results indicate that abnormally high expression of pyroptosis-related genes in epithelial cells, macrophages, and lymphocytes can promote the expression of certain genes, and abnormal expression of these genes activates tumor-related pathways, whereas abnormally high expression of pyroptosis-related genes and regulated genes can affect macrophages and lymphocytes, ultimately affecting tumor formation and progression. Three clusters were identified in the UCEC and control samples based on genes related to pyroptosis. The two most abundant clusters were clustered mainly in epithelial cells and macrophages. We identified highly expressed genes in each cell type and performed a Gene Ontology (GO) analysis using these genes. The results further confirmed the accuracy of the cluster definitions. Taken together, our findings showed a pyroptosis-related gene composition in UCEC and provided a comprehensive representation of the potential pyroptosis effects on UCEC, warranting further study.

### *IL-6* activates tumor-associated pathways in epithelial cells of UCEC

**Figure 3:**
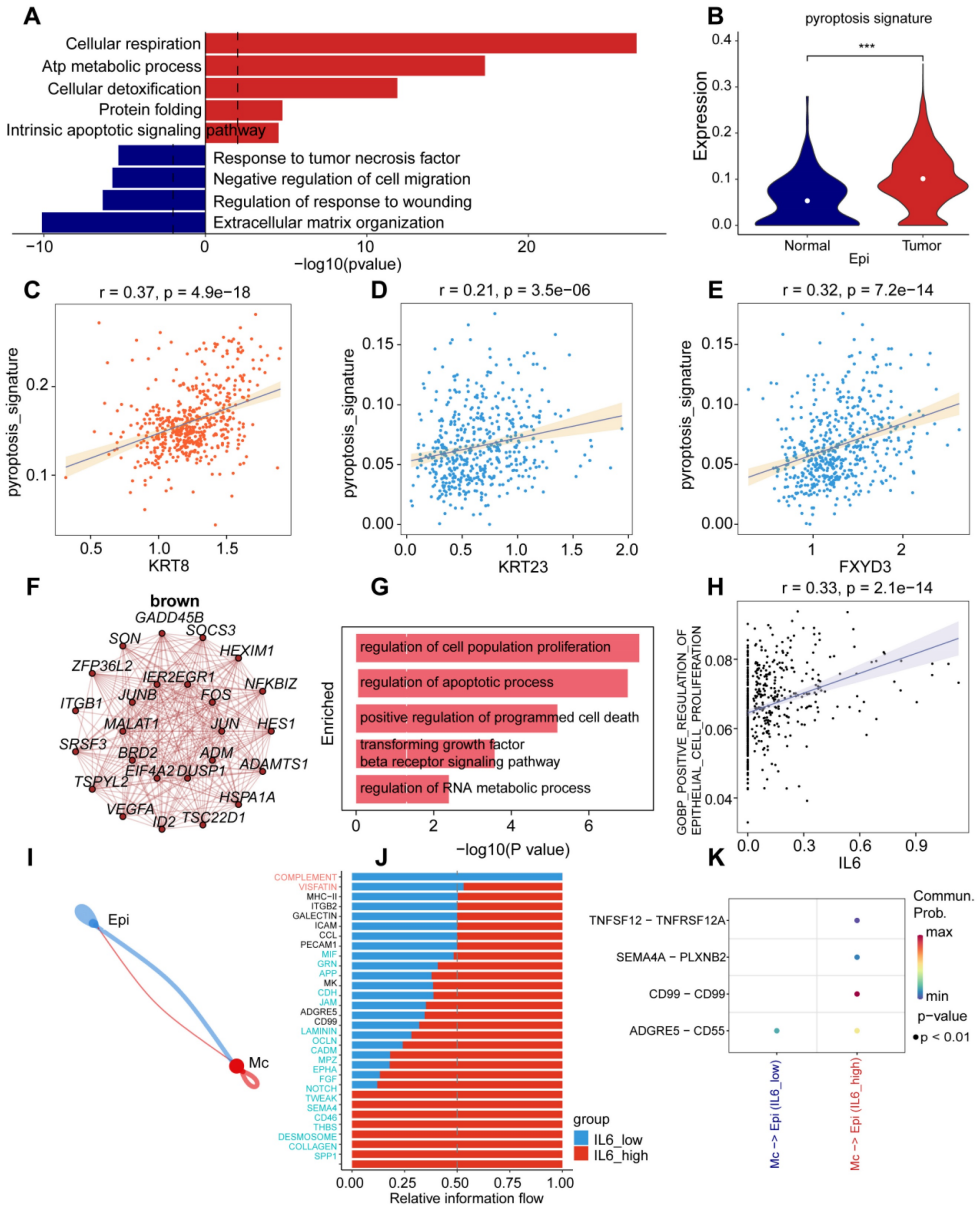
** Specific upregulation of IL-6 in epithelial cells resulted in pathogenesis of UCEC. (A)** Horizontal bar graphs represent the most differentially expressed pathways. Functional enrichment of genes higher (up) and lower (down) was observed in UCEC relative to controls. **(B)** Significant dysregulation of the pyroptosis signature of UCEC in epithelial cells. (Wilcoxon test, *P* < 0.001). **(C)** Correlation between *KRT8* and pyroptosis signature in UCEC.R = 0.37, *P* < 0.05. **(D)** Correlation between *KRT23* and pyroptosis signature in UCEC.R = 0.21, *P* < 0.05. **(E)** Correlation between *FXYD3* and pyroptosis signature in UCEC.R = 0.32, *P* < 0.05. **(F)** Network analysis of the correlation between* IL-6* and other genes. **(G)** Barplot of *IL-6* module enriched functional pathway; the X-axis is -log (p-value), the Y-axis represents the different pathways in which genes are involved. **(H)** Correlation between *IL-6* and the positive regulation of epithelial cell proliferation (R = 0.3,3* P*<0.05, Spearman's correlation analysis). **(I)** Cellchat analysis of the communication between myeloid and epithelial cells. **(J)** The proportion of different gene expressions between the low and high* IL-6* groups. **(K)** Communication and ligand-receptor interaction between myeloid cells and epithelial cells are shown in the dot plot (high *IL-6* versus low *IL*-*6*).

We found that epithelial cells were the most abundant cell population in UCEC, and that the expression of pyroptosis-related genes was highly clustered in this cell type (pyr-1). To further explore the biological functions of epithelial cells, we performed biological function enrichment analysis of DEGs in epithelial cells. Pathways involving upregulated DEGs included cellular respiration, ATP metabolic processes, cellular detoxification, protein folding, and intrinsic apoptotic signaling pathways. The pathways associated with downregulated DEG included the tumor necrosis factor (TNF) pathway, negative regulation of cell migration, regulation of the response to wounding, and extracellular matrix organization (Fig. 3A).

To further explore the role of pyroptosis-related genes in epithelial cells, violin plots exhibited differences in pyroptosis characteristics between the normal and tumor groups using AUCell. The results showed that the pyroptosis signature of the tumor group was higher than that of the normal group in the epithelial cells of UCEC (Fig. 3B). We also analyzed the correlation between identified genes in pyr-1 and the pyroptosis signature in epithelial cells, determined that there was a positive relationship between the pyroptosis signature and* KRT8* (R = 0.37), *KRT23* (R = 0.21), and* FXYD3* (R = 0.32; Fig. 3C-E). These results suggest that abnormally high expression of pyroptosis genes regulates tumor-associated signaling pathways in UCEC epithelial cells.

To identify the pyroptosis-related genes responsible for cell type-specific dysregulation in epithelial cells, we used pyroptosis-related genes concentrated in UCEC to identify the key modules associated with epithelial cells using weighted correlation network analysis (WGCNA), which revealed that 20 genes were closely related to pyroptosis-related gene expression in epithelial cells. In particular, the brown module presented the upregulation of the pyroptosis-related gene *IL-6*. To better understand the functional role of *IL-6*, we searched for 20 genes associated with *IL-6* (Fig. 3F), and GO functional enrichment analyses were performed. The analysis revealed upregulation of cell proliferation, regulation of apoptotic processes, positive regulation of programmed cell death, transforming growth factor (TGF)-β receptor signaling pathway, and regulation of RNA metabolic processes (Fig. 3G). Furthermore, *IL-6* expression positively correlated with epithelial cell proliferation (R = 0.33; Fig. 3H). Next, CellChat was used to examine the interaction between *IL-6* expression in epithelial and other cells (Fig. 3I), and it was observed that the *IL-6* high expression group displayed significant interactions with specific genes (including *NOTCH, TWEAK, SEMA4, CD46, THBS, DESMOSOME, COLLAGEN* and* SPP1*) and other cell types in patients with UCEC (Fig. 3J). Some receptor-ligand pairs were expressed only in the high *IL-6* expression group, such as TNFSF12-TNFRSF12A, SEMA4A-PLXNB2, and CD99-CD99 (Fig. 3K). The above results revealed that *IL-6*, an essential pyroptosis-related gene in epithelial cells, dysregulates epithelial cell proliferation and interferes with the specific regulatory role of receptor-ligand pairs in the epithelial cells of UCEC.

### *BAX* and *TNF* in macrophages promoted tumor progression through the pyroptosis pathway

**Figure 4:**
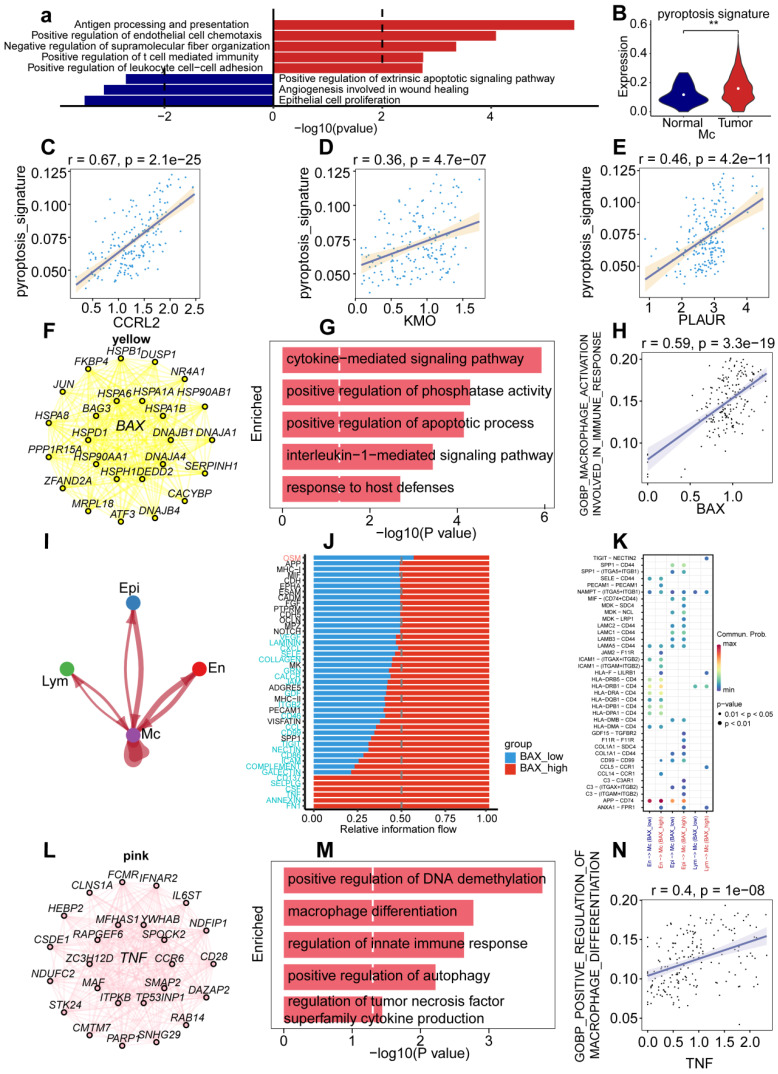
** Specific upregulation of BAX and TNF in macrophages resulted in immune dysfunction of UCEC. (A)** Horizontal bar graphs representing the most differential pathways. Functional enrichment of genes higher (Up) and lower (Down) expressed in UCEC relative to controls. **(B)** Significant dysregulation of the pyroptosis signature of UCEC in macrophages. (Wilcox test, *P* < 0.001). **(C)** Correlation between *CCRL2* and pyroptosis signature in UCEC.R = 0.67, *P* < 0.05. **(D)** Correlation between *KMO* and pyroptosis signature in UCEC.R = 0.36 *P*<0.05. **(E)** Correlation between *PLAUR* and pyroptosis signature in UCEC.R = 0.46, *P* < 0.05. **(F)**Network analysis of the correlation between* BAX* and other genes. **(G)** Barpot of *BAX* module enriched functional pathway; the X-axis is -log (p-value), the Y-axis represents the different pathways in which genes are involved. **(H)** Correlation between *BAX* and macrophage activation involved in immune response (R = 0.59, *P* < 0.05. Spearman correlation analysis). **(I)** Cellchat analysis of communication between lymphocytes, endothelial cells, myeloid cells, and epithelial cells. **(J)** The proportion of different gene expressions between the low and high* BAX* groups. **(K)** Communication and ligand-receptor interactions between endothelial cells and myeloid cells, epithelial cells and myeloid cells, and lymphocytes and myeloid cells, shown in the dot plot (high *BAX* versus low *BAX*). **(L)** Networks of the WGCNA module that included *TNF.* **(M)** Barpot of *TNF* module enriched functional pathway; the X-axis is -log (p-value), the Y-axis represents the different pathways in which genes are involved. **(N)** Correlation between *BAX* and positive regulation of the macrophage immune response (R = 0.59, *P* < 0.05, Spearman correlation analysis).

Because the expression of genes related to pyroptosis was highly clustered in the macrophage group (pyr-2), we performed a biological function enrichment analysis of differentially expressed genes (DEGs) in UCEC macrophages to explore the biological function of macrophages. Some pathways were upregulated, such as antigen processing and presentation, positive regulation of endothelial cell chemotaxis, negative regulation of supramolecular fiber internalization, positive regulation of T cell-mediated immunity, and positive regulation of leukocyte cell-cell adhesion. However, some pathways were downregulated, including regulation of the extrinsic apoptotic signaling pathway and epithelial cell proliferation (Fig. 4A).

To explore the role of genes related to pyroptosis in macrophages, we performed a differential expression analysis of the pyroptosis signature, which showed upregulation of the pyroptosis signature in the tumor group compared to the normal group (Fig. 4B). To identify the correlation between pyroptosis and pyr-2 hypervariable genes of pyr-2 in macrophages, we found a positive relationship between the pyroptosis signature and* CCRL2* (R = 0.67),* KMO* (R = 0.36), and* PLAUR* (R = 0.46; Fig. 4C-E). These results suggest that dysregulation of pyroptosis genes may upregulate tumor-associated genes in UCEC macrophages.

To determine the role of genes related to pyroptosis in macrophage cell type-specific dysregulation, we examined gene expression in macrophages. Among the genes, *BAX* showed the highest upregulation compared with other genes, to elucidate the prognostic value of *BAX* upregulation, we used pyroptosis-related genes concentrated in UCEC to identify the key modules associated with macrophage by WGCNA: the yellow and pink modules presented a significant correlation with the macrophages and included genes *BAX* and* TNF*, respectively. To explore the functional enrichment associated with *BAX* expression, we searched for 20 *BAX*-associated genes (Fig. 4F) and performed Gene Ontology (GO) functional enrichment analysis, which showed that cytokine-mediated signaling pathway, positive regulation of phosphatase activity, positive regulation of apoptotic process, interleukin-1-mediated signaling pathway, and response to host defenses were upregulated in macrophages (Fig. 4G). Furthermore, correlation analysis of *BAX* expression and tumor-associated pathways was performed in macrophages to verify the mechanism of *BAX* upregulation in UCEC macrophages. *BAX* expression positively correlated with the activation of macrophages involved in the immune response (R = 0.59; Fig. 4H). Cell chain analysis using *BAX* revealed various distinct interactions between macrophages and the other three cell types, including epithelial, lymphocyte, and endothelial cells (Fig. 4I). The high* BAX* expression group showed significant interactions with specific genes (including *CD137, SELPLG, CSF, TNF, ANNEXIN,* and* FN1*) and other cell types in patients with UCEC (Fig. 4J). Some receptor-ligand pairs, such as TIGHT-NECTIN2, GDF15-TGFBR2, and F11R-F11R, were expressed only in the high *BAX* expression group (Fig. 4K). The above results revealed that *BAX*, as an essential pyroptosis-related gene in macrophages, dysregulates macrophage proliferation and the specific regulatory role of receptor-ligand pairs in UCEC macrophages.

To explore cell type-specific regulation of *TNF* in macrophages, and the role played by TNF in cell type-specific dysregulation in macrophages, WGCNA was performed to identify the biological functions of *TNF* in UCEC macrophages. WGCNA revealed that 20 genes were closely related to *TNF* expression in macrophages (Fig. 4L). Functional enrichment analysis revealed that the genes were mainly enriched in DNA demethylation, macrophage differentiation, regulation of the innate immune response, positive regulation of autophagy, and regulation of *TNF* superfamily cytokine production (Fig. 4M). Furthermore, a positive correlation was observed between *TNF* and positive regulation of macrophage differentiation (Fig. 4N). Cell chain analysis using *TNF* showed diverse and distinct interactions between macrophages and the other three cell types (Fig. S2A). The samples were then divided into low- and high-TNF expression groups to further explore the role of *TNF* in macrophages.

We found that the *TNF* high-expression group showed significant interactions with specific genes (including *CD137, SELPLG, CSF, TNF,* and* FN1*) and other cell types in patients with UCEC (Fig. S2B). The *TNF* high-expression group showed significant interactions with some receptor-ligand pairs that were expressed, such as TIGHT-NECTIN2, CCL5-CCR1, and CCL14-CCR1 (Fig. S2C). The above results revealed that *BAX* and* TNF*, as essential pyroptosis-related genes in macrophages, can not only specifically dysregulate macrophage differentiation and immune response but also dysregulate receptor-ligand pairs in UCEC macrophages.

### Regulation of pyroptosis-related genes on biological pathways in endothelial cells, fibroblasts, and lymphocytes

To further explore the biological functions of pyroptosis genes in endothelial cells, we performed a biological function enrichment analysis of DEGs within endothelial cells. Several pathways were upregulated, such as those involved in the positive regulation of cell activation, positive regulation of cell adhesion, positive regulation of cytokine production, positive regulation of immune effector processes, and regulation of IL1 production. However, the endothelial cell apoptotic process, vasculogenesis, migration, and differentiation were downregulated (Fig. S3A). To reveal the role of pyroptosis-related genes in endothelial cells, we performed a differential expression analysis of the pyroptosis signature. Violin plots showed upregulation of the pyroptosis signature in the tumor group compared to that in the normal group in endothelial cells (Fig. S3B). These results suggest that dysregulation of pyroptosis genes can upregulate tumor-associated genes in endothelial cells of UCEC. To explore the role of pyroptosis-related genes in cell type-specific dysregulation of endothelial cells, we examined the expression of pyroptosis-related genes in endothelial cells. Among these genes, *CHMP4A* showed the highest upregulation compared to others. To elucidate the prognostic value of *CHMP4A* upregulation, we used pyroptosis-related genes concentrated in UCEC to identify key modules associated with endothelial cells by WGCNA, and discovered that blue modules, including *CHMP4A,* were significantly correlated with endothelial cells (Fig. S3C). Next, we searched for 20 genes associated with *CHMP4A* expression (Fig. S3D) and identified four signaling pathways associated with tumors that were enriched by GO functional enrichment analysis-cellular response to IL1, response to TNF, angiogenesis, wound healing, and cell spreading-which were upregulated in endothelial cells (Fig. S3E). Cell chain analysis using *CHMP4A* revealed diverse and distinct interactions between endothelial cells and the other two cell types, including epithelial cells and macrophages (Fig. S3F). We found that the *CHMP4A* high-expression group displayed significant interactions with specific genes (including *CD137, PROS, GAS, CEACAM, NOTCH, CD46, CSF,* and* FN1*) and other cell types in patients with UCEC (Fig. S3G), Some receptor-ligand pairs were expressed only in the high *CHMP4A* expression group, such as LGALS9-CD44, LAMC2-CD44, LCMC2-(ITGA9+ITGB1), LCMC1-(ITGA9+ITGB1), and LCMB3-(ITGA9+ITGB1; Fig. S3H). These results revealed that *CHMP4A* is an essential pyroptosis-related gene in endothelial cells that dysregulates the regulation of endothelial cell proliferation and the specific regulatory role of receptor-ligand pairs in endothelial cells of UCEC.

To further explore the biological functions of pyroptosis genes in fibroblasts, we performed a biological function enrichment analysis of DGEs within fibroblasts. Some pathways, such as wound healing, collagen fibril organization, intrinsic apoptotic signaling, response to hypoxia, and response to TGF-β, were upregulated. However, other genes were downregulated, including those involved in negative regulation of the cell cycle, regulation of transferase activity, and regulation of cell proliferation (Fig. S4A). Violin plots showed upregulation of the pyroptosis signature in the tumor group compared with that in the normal group of fibroblasts (Fig. S4B). These results suggest that the dysregulation of pyroptosis genes can regulate tumor-associated genes in UCEC fibroblasts. To explore the role of pyroptosis-related genes in the cell type-specific dysregulation of endothelial cells, the expression of pyroptosis-related genes in endothelial cells was examined. Among the genes, *BAX* showed higher upregulation compared to others, and to elucidate the prognostic value of *BAX* upregulation, we used pyroptosis-related genes concentrated in UCEC to identify key modules associated with endothelial cells by WGCNA, discovering purple modules that were significantly correlated with endothelial cells. The turquoise module genes were *BAX and COL6A3,* respectively. (Fig. S4C). Next, we searched for 20 *BAX*-associated genes (Fig. S4D). Five signaling pathways associated with tumors were enriched in GO functional analysis, including extracellular matrix organization, collagen fibril organization, regulation of apoptotic processes, cellular response to TGF-β stimulus, and positive regulation of fibroblast proliferation (Fig. S4E). Cell chain analysis using *BAX* showed various distinct interactions between fibroblasts and four other cell types: epithelial cells, lymphocytes, endothelial cells, and macrophages (Fig. S4F). We found that the high* BAX* expression group showed significant interactions with specific genes (including *WNT, ANGPTL, ncWNT,* and* THY1*) and other cell types in patients with UCEC (Fig. S4G). Some receptor-ligand pairs, such as D99-CD99, ADGRE5-CD55, and SPP1-CD44, were expressed only in the high *BAX* expression group (Fig. S4H). The above results revealed that *BAX is* an essential pyroptosis-related gene in fibroblasts and dysregulates endothelial cell proliferation and the specific regulatory activity of receptor-ligand pairs in UCEC fibroblasts.

To further explore the biological function of pyroptosis genes in lymphocytes, we utilized WCGNA to identify key pyroptosis genes in lymphocytes; the key gene was *TNF*. We analyzed the correlation of other genes that were expressed within lymphocytes with the pyroptosis signature and found that there was a positive relationship between *PTPCR* and the pyroptosis signature (Fig. S5A).

Next, we searched for 20 genes associated with *TNF* (Fig. S5B), the five signaling pathways associated with tumors were enriched by GO functional analysis and included positive regulation of cytokine production, I-kappa B kinase/NF-kappa B signaling, regulation of T cell activation, regulation of the T cell-mediated immune response to tumor cells, and cell response to tumor necrosis factor (Fig. S5C). Cellchat analysis using *TNF* showed various distinct interactions between lymphocytes and the other three cell types, including epithelial cells, endothelial cells, and macrophages (Fig. S5D). Next, we divided the samples into low- and high-TNF expression groups to further explore the role of *TNF* in lymphocytes. The receptor-ligand pairs of the two groups were not different (Fig. S5E). These results revealed that *TNF*, the most essential gene related to pyroptosis in lymphocytes, could activate tumor-related pathways in UCEC lymphocytes.

### Pyroptosis-related genes inhibit immune cell infiltration and hinder immune pathway activation

**Figure 5:**
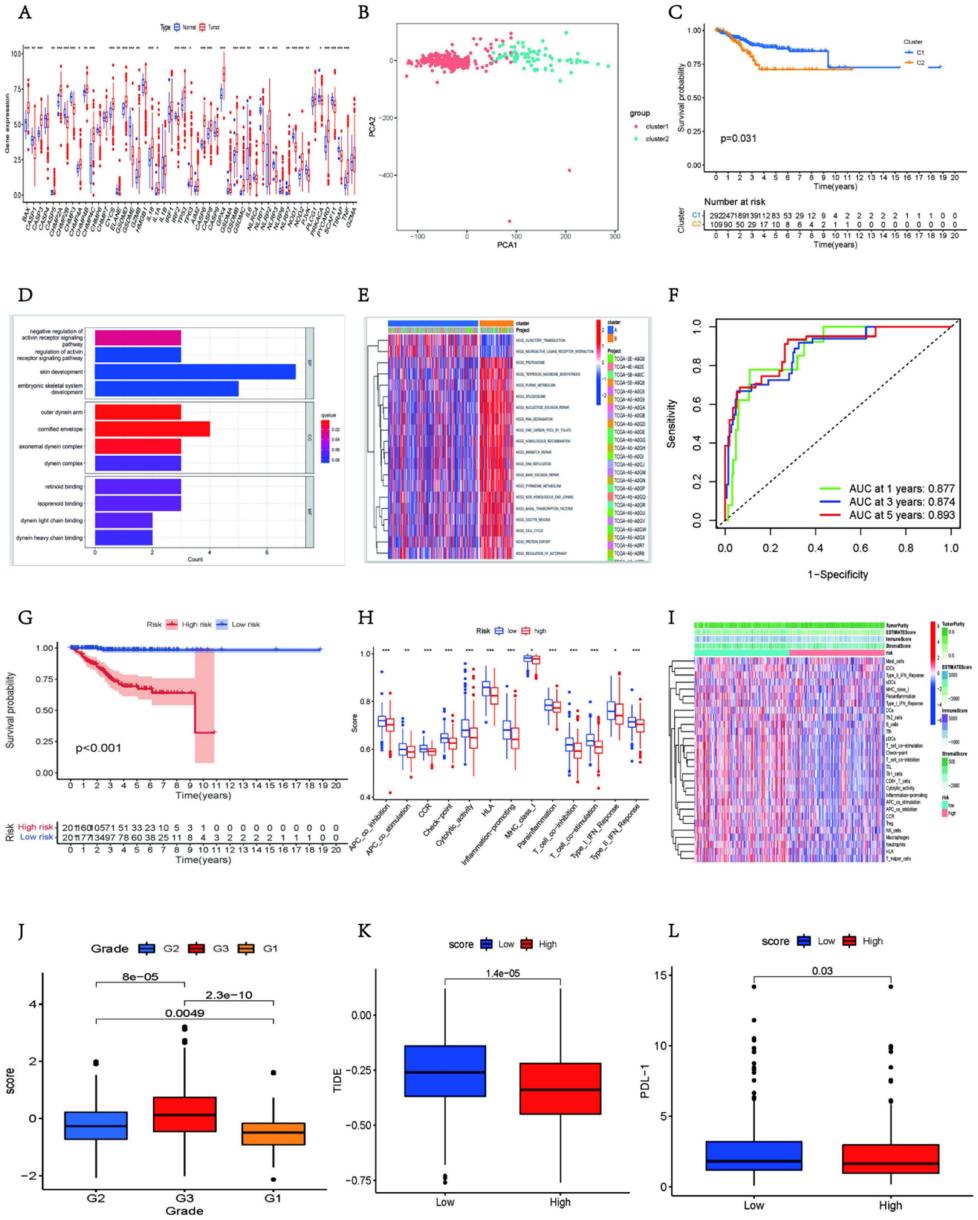
** Pyroptosis score for establishing a risk prognostic model via RNA-seq. (A)** Difference in gene expression levels of 51 pyroptosis regulators between normal and UCEC samples. The upper and lower ends of the boxes represent the interquartile ranges of the values. The lines in the boxes represent the median values and the dots show outliers. Asterisks represent the statistical P-values (*P < 0.05; ** *P* < 0.01; *** *P* < 0.001). **(B)** PCA plot of patients in different groups. **(C)** Kaplan-Meier curves for the OS of patients in cluster1 and cluster2 (two-stage test, *P* < 0.05). **(D)** The bar plot shows significant GO functional items. **(E)** GSVA enrichment analysis showing the activation states of the biological pathways in distinct pyroptosis regulator clusters. The heatmap was used to visualize these biological processes; red represents activated pathways, and blue represents inhibited pathways. The UCEC cohorts were used as sample annotations. **(F)** Time-dependent receiver operating characteristic curves assessing the prognostic performance of the risk score in the UCEC cohort. **(G)** Survival probability of patients in the high-risk and low-risk groups in the UCEC cohorts displayed by Kaplan-Meier curves. **(H)** Boxplots depicting cell immune response differences between the two groups. **(I)** Heatmap showing the activation states of immune functions and immune cells in distinct score groups, and tumor purity, ESTIMATE score, immune score, and stromal score were used as indicators of immune function. Red, activated state; blue, inhibition state. **(J)** Differences in risk scores among different grade groups. **(K-L)** Difference in TIDE (K) and PDL-1 (L) between high-risk and low-risk groups, respectively.

To further verify the role of pyroptosis-related genes in tumor microenvironment (TME) and their clinically relevant characteristics, we used data from The Cancer Genome Atlas (TCGA) to analyze the transcriptome data of 401 patients with UCEC based on pyroptosis-related genes. Analysis of the copy number variation (CNV) frequency in genes related to pyroptosis revealed copy number amplification (Fig. S6A). Furthermore, there were some interactions between the genes related to pyroptosis (Fig. S6B). The heatmap showed higher expression of most pyroptosis-related genes in the tumor group than in the normal group (Fig. 5A).

These results suggest that pyroptosis-related genes play vital roles in the onset and development of UCEC.

Unsupervised cluster analysis and principal component analysis (PCA) classified 401 patients with UCEC and identified two distinct modification patterns (Figs. S6C and 5B), including 292 cases with pattern A and 109 cases with pattern B. These clusters were named clusters 1 and 2, respectively. Survival analyses indicated that these clusters were significantly associated with the prognosis of patients with UCEC, with cluster 1 exhibiting a prominent survival advantage (Fig. 5C). Next, we performed functional enrichment analysis using all genes. GO enrichment analysis revealed that certain tumor-related pathways were activated, such as the regulation of the activin receptor signaling pathway, outer dynein arm, cornified envelope, and axonemal dynein complex (Fig. 5D). Kyoto Encyclopedia of Genes and Genomes (KEGG) enrichment analysis showed that compared to cluster 1, pathways related to DNA replication and nucleotide repair were expressed higher in cluster 2 (Fig. 5E).

Because the above analyses were based on the entire cohort, they did not accurately predict the patterns of pyroptosis-related expression in individual tumors. Therefore, a prognostic risk-scoring model was established using least absolute shrinkage and selection operator (LASSO) regression analysis to quantify pyroptosis-related gene expression in individual patients with UCEC and to predict the response to treatment and prognosis of patients with UCEC. We selected genes related to pyroptosis with a non-zero coefficient and used them in the survival analysis (Fig. S6D). Receiver operating characteristic (ROC) curves validated the predictive power of this prognostic risk model (Fig. 5F). PCA of pyroptosis-related genes with non-zero coefficients stratified the patients into two groups (Fig. S6E). The best cut-off value was selected, and patients with scores above this value were defined as the pyroptosis high-score group; otherwise, they were defined as the pyroptosis low-score group (Fig. S6F). Survival analysis revealed better survival in the low-scoring group (Fig. 5G).

To investigate the role of pyroptosis-related genes in immune cell infiltration of the TME, we compared the differences in the degree of immune cell infiltration and immune-related pathway status between the two groups. We found that in the pyroptosis high-score group, immune cells, including B cells, T cells, NK cells, and macrophages, were less represented (Fig. S6g), and immune-related pathways were also inhibited in this group, such as type I/II IFN response, T cell co-stimulation, and inflammation-promoting pathways (Fig. 5h). The heatmap more intuitively reveals that a variety of immune cells and immune pathways present low expression states in the high-score group (Fig. 5I). The microenvironment, stroma, immune system, macrophages, and ESTIMAT scores were lower in the high-score group than in the low-score group (Figs. S6H-L). However, tumor purity was higher in the high-scoring group (Fig. S6M). These results indicate that genes related to pyroptosis can block the infiltration of immune cells and inhibit the related immune pathways.

### Pyroptosis-related genes can predict clinical characteristics and treatment

To better validate the assessment potential of pyroptosis-related gene scores in patients with UCEC, we combined this score with associated clinical characteristics, including age, tumor grade, and survival. We observed the proportion of patients with different clinical features in the high- and low-scoring groups. The histogram shows that in the high-score group, the proportion of patients with tumor grade 3, death status, and age >65 years was higher (Figs S6N-P). Additionally, the box plots more intuitively showed the difference in scores between the different clinical groups; patients in the G3 group had significantly higher scores than those in the G1 and G2 groups (Fig. 5J), and the scores were also higher in the group of patients who died and were >65 years of age (Figs. S7A-B). Survival analysis also showed that survival in the low group was always better than that in the high group, regardless of age and tumor grade (Figs. S7C-F). These results demonstrate that the score could predict individual characteristics and has the potential to serve as a biomarker to assess clinical characteristics and predict prognosis in patients with UCEC.

To verify how the score is related to anticancer drug responses, we used Tumor Immune Dysfunction and Exclusion (TIDE) to predict the therapeutic effects of ICI based on tumor profiles prior to treatment. The TIDE score and PD-1 expression were significantly higher in the low-score group than in the high-score group (Figs. 5K-L), indicating that tumors in patients in the low-score group were more likely to experience immune escape and showed less therapeutic effects in response to ICI. However, the IC_50_ values in the low-score group were lower than those in the high-score group for vinblastine, lenalidomide, and temsirolimus (Figs. S7G-J). The results showed that patients with a low UCEC score had a better therapeutic response to anti-tumor drugs.

In summary, genes related to pyroptosis can be used as biological markers to predict the clinical characteristics and treatment effects in individual patients.

### The expression of pyroptosis-related genes was increased in endometrial tumor tissues

To verify the accuracy and consistency of the aforementioned results, we collected three sets of scRNA-seq data for UCEC from the Gene Expression Omnibus (GEO) database. Using UMAP plots, we visualized the cell types in these datasets, which primarily included seven types of cells (myeloid cells, endothelial cells, fibroblasts, smooth muscle cells, Mast/BC, T/NK, and epithelial cells), annotated using classical marker genes (Fig S8A). To better understand the proportions of pyroptosis-related genes, we explored the expression ratios of several key pyroptosis-related genes across different UCEC cell types, finding that *CHMP2A, CHMP3, CHMP4A*, and *BAX* had relatively high expression ratios (Fig S8B). Given the enhanced expression of these pyroptosis-related genes, we performed dimensionality reduction to explore heterogeneity within the cell populations, identifying five distinct subpopulations. To characterize the cell types within each cluster, we analyzed the expression profiles of various pyroptosis-related genes, finding that: Pyr-0 was characterized by *F2RL1*, *IGLC3, APOBEC3G, LYAR, CD6*, and *RASSF8*; Pyr-1 showed prominent expression of *RASSF8, CDH11, MFSD2A, PRLR*, and *WNT16*; Pyr-2 was marked by *ADGRF5, TMEM70, BCL6B, ADGRL4*, and *LDLR*; Pyr-3 also displayed expression of *ADGRF5, TMEM70, BCL6B, ADGRL4,* and *LDLR*; Pyr-4 featured *CTXN1, DPCD, EPPIN, ANKRD66*, and *MOSPD1*; Pyr-5 was distinguished by *PDE4DIP, PILRA, PLA2G7, CASP1*, and *KMO* (Fig S8C).

**Figure 6:**
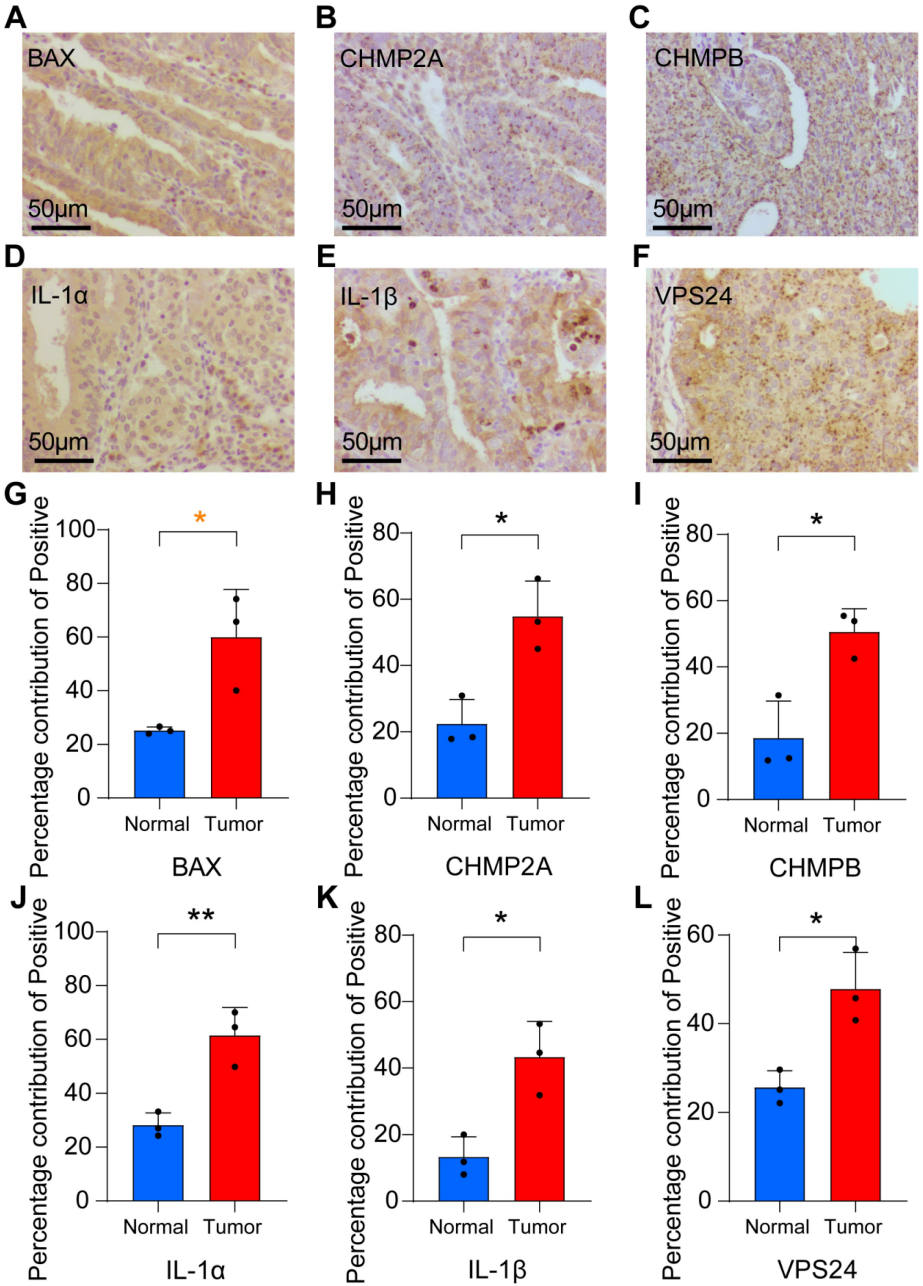
** Immunohistochemistry was used to verify the expression of pyroptosis gene in endocarcinoma. (A-F)** Immunohistochemical analysis was performed on UCEC tissues on endometrial tissues of tumor (n = 18; Scale bars: 50.0 µm). **(G-L)** The percentage contribution of positive staining between normal and tumor groups (*, *P* < 0.05; **, *P* < 0.01; ***, *P* < 0.001; ****, *P* < 0.0001; ns, non-significant).

To further validate whether the expression of the pyroptosis-related gene set was generalized at the molecular level in tissues of patients with UCEC, immunohistochemistry and quantitative Real-Time Polymerase Chain Reaction (RT-qPCR) experiments were performed on endometrial tissues of three patients with UCEC (including endometrial tumor and paracancerous tissues) and three healthy individuals (normal endometrial tissues). We found that, compared to normal tissues, *BAX*, *CHMP2A*, *CHMPB*, *IL1A*, *IL1B,* and *VPS24* were more strongly expressed in the cytoplasm and nucleus of tumor tissues (Figs. 6A-F, S9A-F).

**Figure 7:**
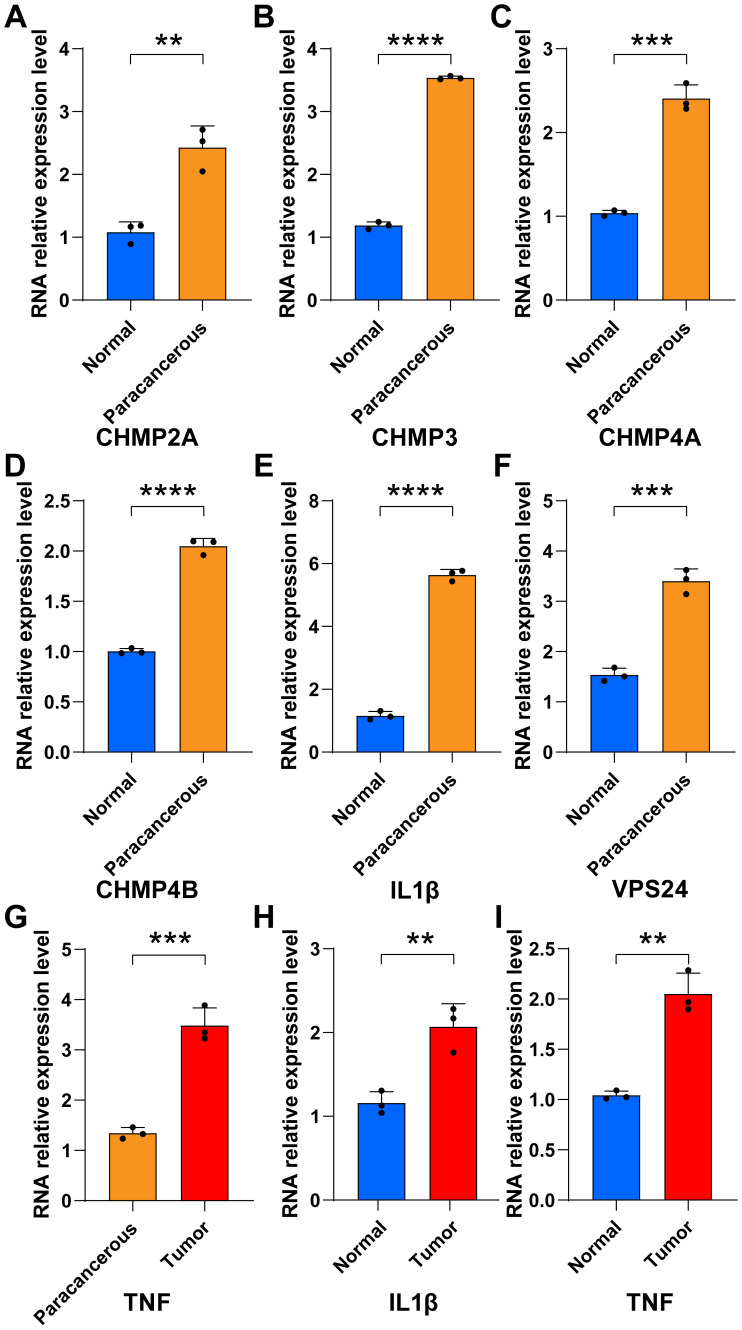
** PCR were used to verify the expression of pyroptosis gene in endocarcinoma. (A-I)** Relative RNA expression levels between normal and paracancerous groups (UCEC tissues from control patients (n = 3) and those with UCEC (n = 3); * *P* < 0.05; ** *P* < 0.01; *** *P* < 0.001; ns, non-significant).

The bar chart shows that in tumor tissues, the percentage contribution of positive genes was higher than that in the normal group (Figs. 6G-L). We also performed RT-qPCR on the six samples. The results showed that in the paracancerous group, the expression of *CHMP2A*, *CHMP3*,* CHMP4A*, *CHMP4B*, *IL1B,* and *VPS24* was higher than that in normal endometrial tissue (Figs. 7A-F).

*TNF* expression was higher in the tumor group as compared to the paracancerous group (Fig 7G). *IL-1B* and *TNF* expression were higher in the tumor group compared to the normal group (Fig 7H-I). These results showed that pyroptosis-related gene expression levels gradually increased from normal to tumor tissues, further verifying that pyroptosis-related genes can act as pro-oncogenic genes to regulate the occurrence and development of UCEC.

## Discussion

Pyroptosis is a type of programmed necrotic cell death activated by intracellular infection with bacteria, viruses, fungi, or protozoa in the presence of pathogen-associated molecular patterns (PAMPs) or cell-derived damage-associated molecular patterns (DAMPs) 26. It is normally induced in cells of the innate immune system, such as monocytes, macrophages, and dendritic cells. Previous studies have shown that the prognosis of patients with malignant tumors is associated with the expression of pyroptosis genes 5; however, the mechanism of pyroptosis-related genes in different cells of UCEC is still unclear.

In this study, cell type-specific upregulation of pyroptosis-related genes exerted distinct functions in the five cell types during the progression of UCEC at the single-cell level 12. Carcinogenesis induces the upregulation of *IL-6* expression in epithelial cells in patients with UCEC 16. We found that *IL-6* plays a role in the dysregulation of cell population proliferation and apoptosis, indicating that* IL-6* may be specific to epithelial cell phenotypes in UCEC and may be a useful therapeutic target. Additionally, *BAX* expression was highly upregulated during macrophage dysfunctional differentiation and immune response in patients. Furthermore, *TNF* upregulates the *TNF* pathway in the macrophages of UCEC 27. Thus, dysregulation of pyroptosis-related genes induces aberrant cell-cell communication in various cell types and mediates ligand-receptor interactions in various cell types in lung tissues by activating the *TNF* signaling pathway, which promotes carcinogenesis. Finally, we designed a risk score related to pyroptosis using a COX regression model with transcriptome information of patients with UCEC derived from a public database and combined this score with clinical features, immune infiltration of the TME, expression of immune checkpoint regulators, and drug sensitivity to further verify the regulatory role of pyroptosis-related genes in UCEC 28. Taken together, these results confirm the diverse potential of pyroptosis genes for prognostic stratification of patients with UCEC.

Endometrial malignancy is one of the three most common gynecological malignancies, and current chemoradiotherapy methods do not improve the 5-year survival rate of patients 3. Therefore, the identification of effective regulatory genes is an important and time-consuming process. Limited research is available on the regulatory mechanisms of pyroptosis genes in UCEC at the single-cell level 9. In this study, the cell type-specific upregulation of pyroptosis genes played distinct roles in the five cell types. In epithelial cells, the upregulation of genes related to pyroptosis induces the activation of intrinsic signaling pathways, such as Cellular respiration, ATP metabolic proces pathways, which can stimulate cell respiration and adenosine triphosphate (ATP) production, and enable tumor cells to meet the energy requirements for growth and metastasis. *IL-6* expression can activate cell proliferation, apoptosis, and RNA metabolism pathways and plays a regulatory role in UCEC 29. In macrophages, upregulation of pyroptosis-related genes, mainly *BAX* and *TNF*, can induce the processing and presentation of antigens, activate T cells and *TNF* signaling pathways, increase immune cell infiltration in the immune microenvironment of tumor patients, and strengthen the natural immune response of the host. Simultaneously, it can induce epithelial cell proliferation. Endothelial cells are chemotactic and work together with differentiated macrophages to regulate UCEC. In endothelial cells, the upregulation of genes related to pyroptosis, mainly dominated by *CHMP4A*, activates several pathways, for example, tumor-associated necrosis factor, induces angiogenesis, wound healing and so on, to promote tumor formation and metastasis 30. In fibroblasts, the upregulation of *BAX* gene expression can activate the formation of TGF-β and the extracellular matrix and promote immune cell infiltration of UCEC 31. In lymphocytes, upregulation of *TNF* gene expression can be mediated by T-cell activation, which regulates the immune response of tumor cells 32. This study focused on the regulatory mechanisms of pyroptotic genes in different endometrial cell types in patients with UCEC. However, the mechanisms by which cell type-specific pyroptosis genes regulate immune cells and related pathways in UCEC must be verified using *in vitro* assays.

In this study, the specific upregulation of pyroptosis genes altered cell-cell communication between the five cell types. In epithelial cells and fibroblasts, activation of CD99-related ligands can inhibit endothelial barrier invasion and migration activity of the endothelial barrier 33; at the same time, in epithelial cells, the SEMA4A-PLXNB2 ligand receptor for activation of immune cells, such as T cells, B cells, and the CCL 5-CCR 1 ligand receptor in macrophages can direct chemotactic T cells, NK cells, and other immune cells, and in turn, activation of these ligand receptors enhances the immune response to tumors and plays a certain role in tumor metastasis 34. TIGHT-NECTIN2 in macrophages serves as an immune checkpoint in the TME, and its interaction can inhibit the function of immune cells in the microenvironment and weaken the immune response, thus promoting tumor infiltration 35. The F11R-JAM 3 ligand receptor in endothelial cells participates in the regulation of various biological processes that regulate tumorigenesis, including paracellular permeability, leukocyte transendothelial migration, epithelial-stromal transformation, and angiogenesis 36. ADGRE5-associated ligand-receptor pairs in fibroblasts can function during leukocyte recruitment 37, activation, and migration, whereas SPP1-associated ligand-receptor pairs can suppress immune cell infiltration in the TME 9. These results further illustrate the overexpression of genes related to pyroptosis, which activates tumor-associated receptor ligand pairs and affects UCEC by affecting immune cells or biological pathways in the TME.

We then used the transcriptome information of UCEC samples from public databases and combined it with the COX model to establish pyroptosis-related risk scores to verify the role of pyroptosis genes in individual UCEC. We combined this score with tumor clinical characteristics, the TME, and immune checkpoints and found that the tumor group had higher scores and the higher the score, the lower the degree of immune cell infiltration; however, the higher the tumor grade, the higher the tumor purity 7. The analysis of immune checkpoints and drug sensitivity showed that the higher the score, the easier the occurrence of immune escape. The effect of treatment on ICI response is good; however, the effect of anti-tumor drugs is poor. These results further illustrate that pyroptosis-related genes can predict individual characteristics and can be used as biological indicators of treatment efficacy in patients with UCEC.

This study provides insights into the mechanisms underlying the regulation of pyroptosis-related genes in UCEC. In this study, pyroptosis genes were upregulated in UCEC tissues and played an important role in immune dysregulation, activation, and the development of intrinsic signaling pathways. Induced changes in cell-cell communication may lead to functional disorders of different cells in the endometrium. The upregulation of pyroptosis genes in different cell types is of prognostic significance and supports the development of drug-activating genes for pyroptosis.

As the above studies were based on theoretical studies, we supplemented them with RT-qPCR and immunohistochemistry. Immunohistochemically, the positive rate of HOX expression was significantly increased in tumor tissues. RT-qPCR verified that in endometrial tumor tissues, HOX expression was higher than that in the normal group, which further confirmed the theoretical studies above.

In conclusion, we conducted a comprehensive analysis of pyroptosis-related genes across five cell types in UCEC, demonstrating their predictive capabilities for UCEC. The pyroptosis prognostic risk model also showed an independent prognostic ability, providing novel and insightful perspectives for our research field. In the future, pyroptosis-related genes could serve as cutting-edge areas for unique diagnostic markers and prognostic indicators in UCEC, offering important information for enhancing early diagnosis and guiding more precise treatment strategies.

## Supplementary Material

Supplementary figures and tables.

## Figures and Tables

**Table 1:** Comparison of basic data from eligible selected endometrial cancer patients and controls. Including age, tumor size, histology, histological grading, FIGO staging, lymph node metastasis and treatment method.

Patient		Ca1	Ca2	Ca3	N1	N2	N3
**Characteristic**	**Age**	58	56	67	46	54	61
	**Tumor size (cm)**	2.5*2.1*1.3	1.5*1.0*0.5	3.0*2.0*1.6	-	-	-
	**Histology**	Adenocarcinoma	Adenocarcinoma	Adenocarcinoma	-	-	-
	**Histological grading**	G1	G1	G2	-	-	-
	**Bokhman typing**	I	I	I	-	-	-
	**FIGO staging**	II	ⅠA	ⅢC1	-	-	-
	**Lymph node metastasis**	negative	negative	negative	-	-	-
	**Treatment method**	Surgery Radiotherapy	surgery	Surgery Chemotherapy Radiotherapy	-	-	-

## Abbreviations

UCEC: Endometrial cancer
scRNA-Seq: single-cell RNA sequencing
TNF: tumor necrosis factor
PCR: Quantitative real-time
TME: tumor microenvironment
IFN: interferon
DAMPs: damage-associated molecular patterns
WGCNA: Weighted correlation network analysis
TGF: transforming growth factor
DEGs: differentially expressed genes
GO: Gene Ontology
TCGA: The Cancer Genome Atlas
CNV: copy number variation
PCA: principal component analysis
KEGG: Kyoto Encyclopedia of Genes and Genomes
LASSO: east absolute shrinkage and selection operator
ROC: receiver operating characteristic
RT-qPCR: quantitative Real-Time Polymerase Chain Reaction
ATP: Adenosine triphosphate
TIDE: Tumor Immune Dysfunction and Exclusion
ssGSEA: single-sample gene-set enrichment analysis
CAFs: cancer-associated fibroblasts
scRNA-seq: Single-cell RNA sequencing
CXCL: Chemokine (C-X-C motif) ligand
PAMPs: Pathogen-associated molecular patterns
UMAP: uniform manifold approximation and projection
UMI: unique molecular identifier
