# Supplementary material for: Cell-type-specific regulators landscape and regulatory mechanisms underlying pyroptosis in uterine corpus endometrial carcinoma
Source: J Cancer. 2025 Jan 1;16(2):660–79. doi: 10.7150/jca.100547 (PMC11685693; doi:10.7150/jca.100547)
Supplement: Supplementary file 1 — Supplementary figures and tables. [file jcav16p0660s1.pdf]

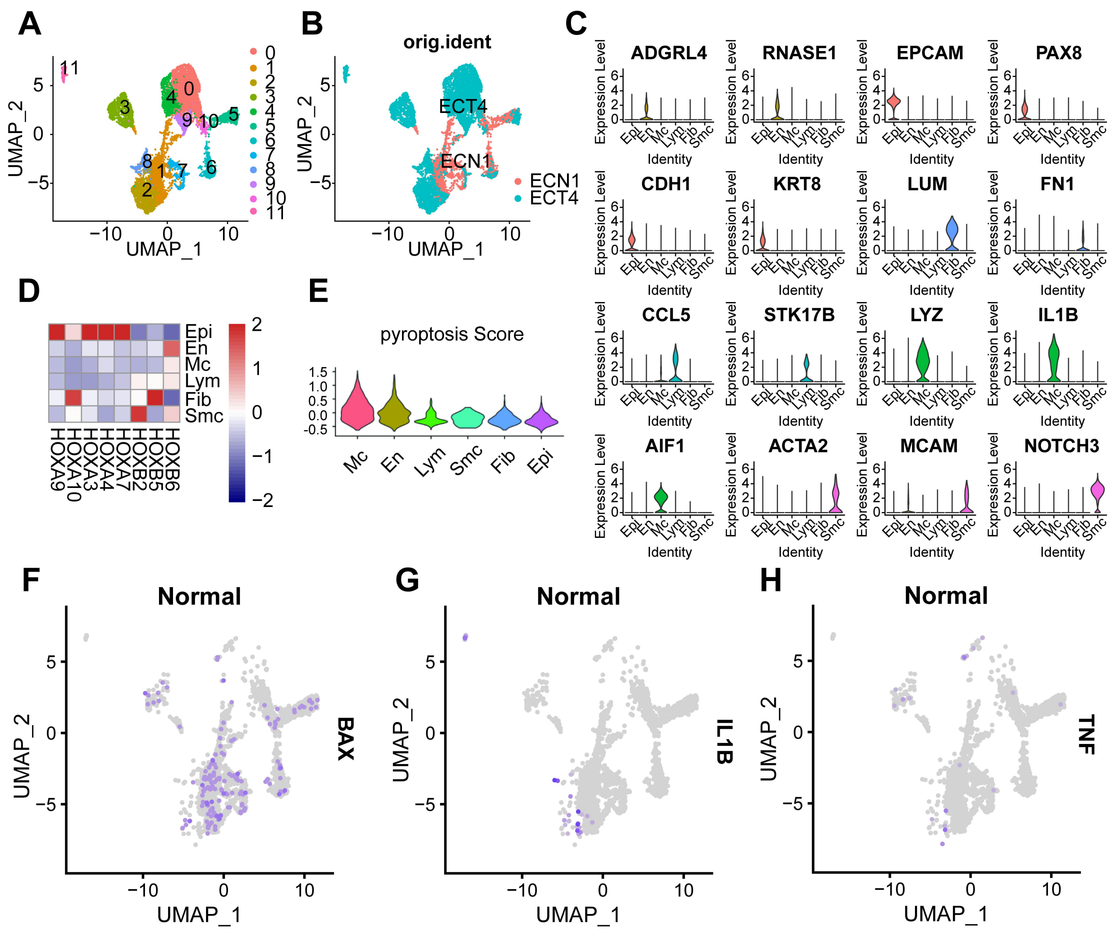

Fig. S1 (A) UMAP for dimensionality reduction and identified clusters by pyroptosis regulators, showing 11 clusters. (B) UMAP for dimensionality reduction and identified clusters by pyroptosis regulators, showing 2 clusters. (C) Sixteen genes were expression level in five cell types. (D) Heatmap of nine genes by five cell from relationship. (E) pyroptosis score in each cell type. (F-H) Mean expression levels of the *BAX* (F), *IL1B* (G) and *TNF* (H) in normal tissue.

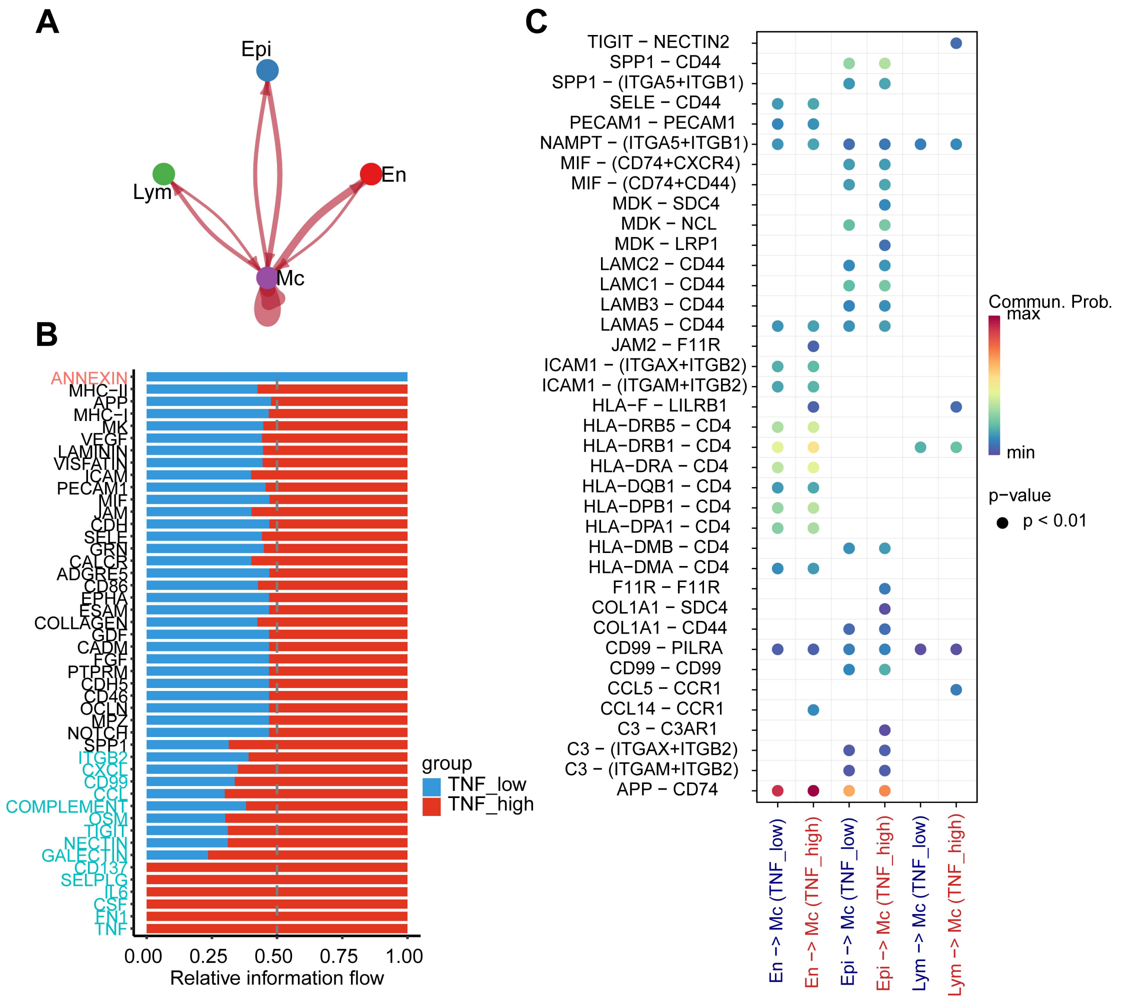

Fig. S2 (A) Cellchat analysis of the communication between four kind of cells. (B) The proportion of different gene expression between the low and high *TNF* groups. (C) Communication and ligand-receptor interaction between Endothelial cells and myeloid cells,epithelial cells and myeloid cells , Lymphocyte and myeloid cells, showing in the dotplot (high *TNF* versus low *TNF*).

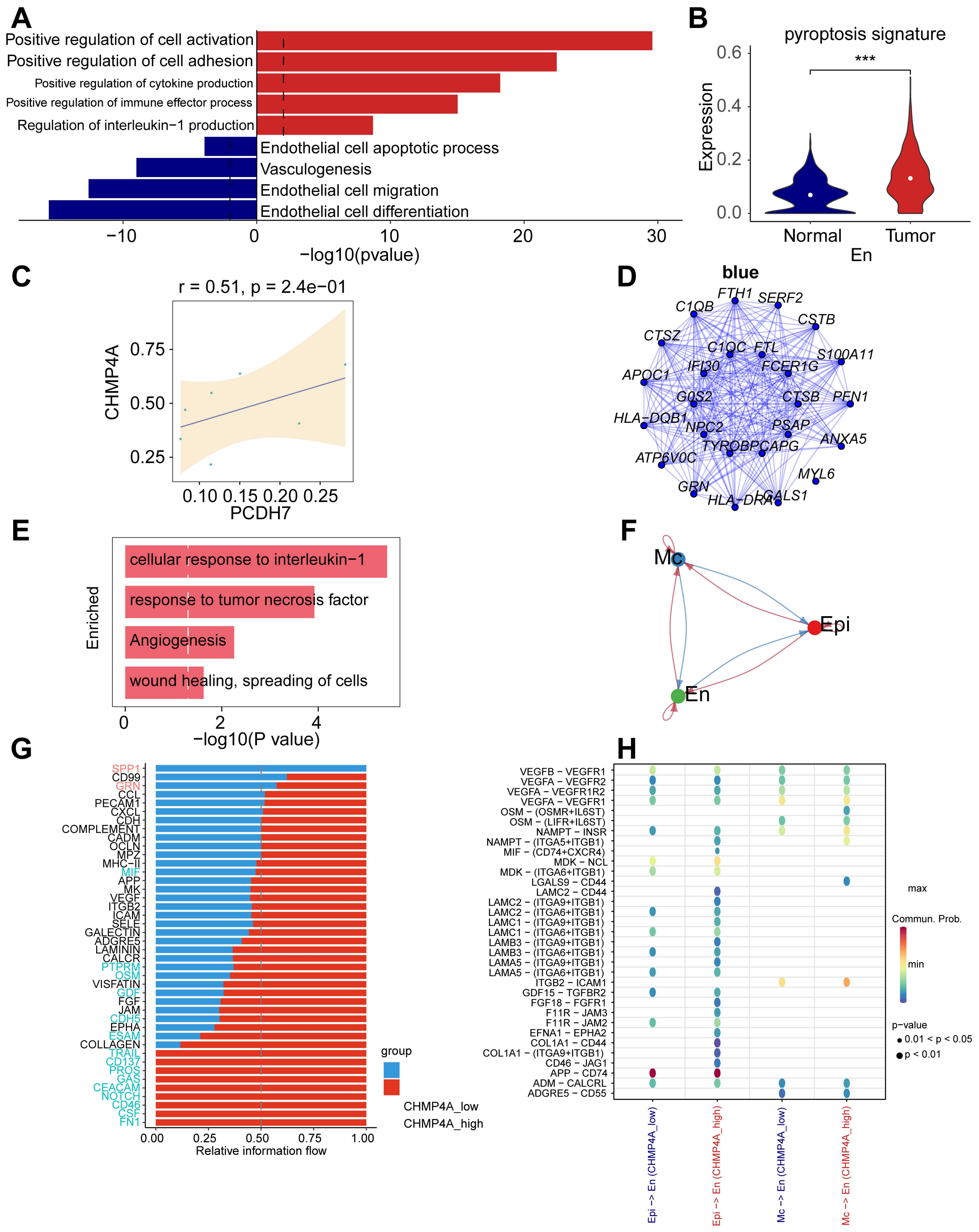

Fig. S3 (A) Horizontal bar graphs representing the most differential pathways. Functional enrichment of genes higher (Up) and lower (Down) expressed in UCEC relative to controls. (B) Significant dysregulation of pyroptosis signature of UCEC in epithelial cells. (Wilcox test, p<0.001). (C) Correlation between the *CHMP4A* and *PCDH7* in UCEC.R=0.31 p < 0.05. (D) Network analysis of the correlation between

CHMP4A and other genes. (E) Barplot of *IL-1B* module enriched function pathway; the X-axis is -log (p-value), the Y-axis is the different pathways in which genes are involved. (F) Cellchat analysis of the communication between epithelial cells, myeloid cells and epithelial cells. (G) The proportion of different gene expression between the low and high *CHAM4P* groups. (H) Communication and ligand-receptor interaction between Endothelial cells and Fibroblast cells, epithelial cells and Fibroblast cells, Lymphocyte and Fibroblast myeloid cells and Fibroblast cells, showing in the dotplot (high *CHMP4A* versus low *CHMP4A*).

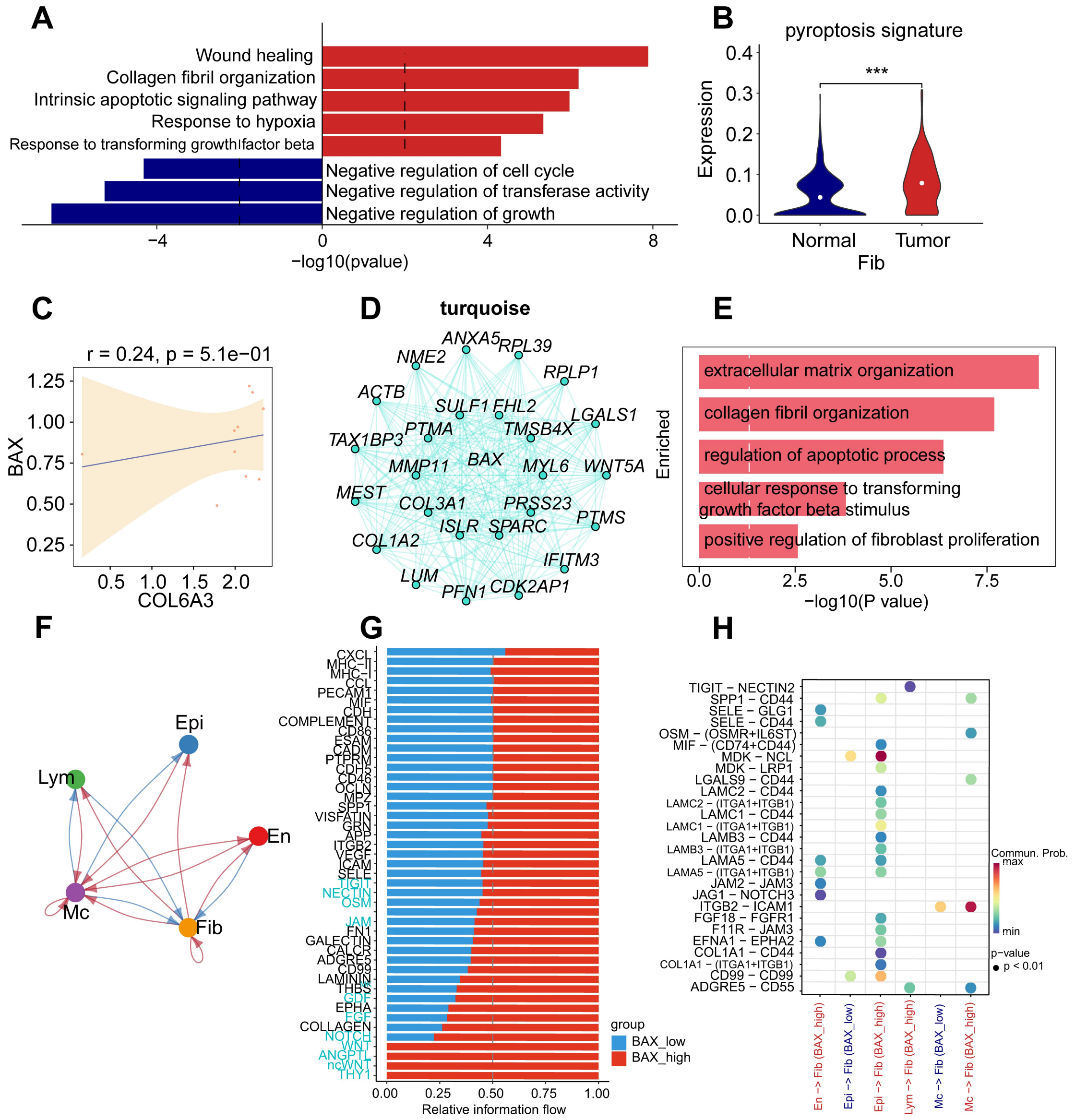

Fig. S4 (A) Horizontal bar graphs representing the most differential pathways. Functional enrichment of genes higher (Up) and lower (Down) expressed in UCEC relative to controls. (B) Significant dysregulation of pyroptosis signature of UCEC in epithelial cells. (Wilcox test, p<0.001). (C) Correlation between the *BAX* and *COLBA3* in UCEC.R=0.24 p < 0.05. (D) Network analysis of the correlation between *BAX* and other genes. (E) Barplot of *BAX* module enriched function pathway; the X-axis is -log (p-value), the Y-axis is the different pathways in which genes are involved. (F) Cellchat analysis of the communication between five kind of cells (G)

The proportion of different gene expression between the low and high *BAX* groups. (H) Communication and ligand-receptor interaction between Endothelial cells and Fibroblast cells ,epithelial cells and Fibroblast cells, Lymphocyte and Fibroblast myeloid cells and Fibroblast cells, showing in the dotplot (high *IL-6* versus low *IL-6*).

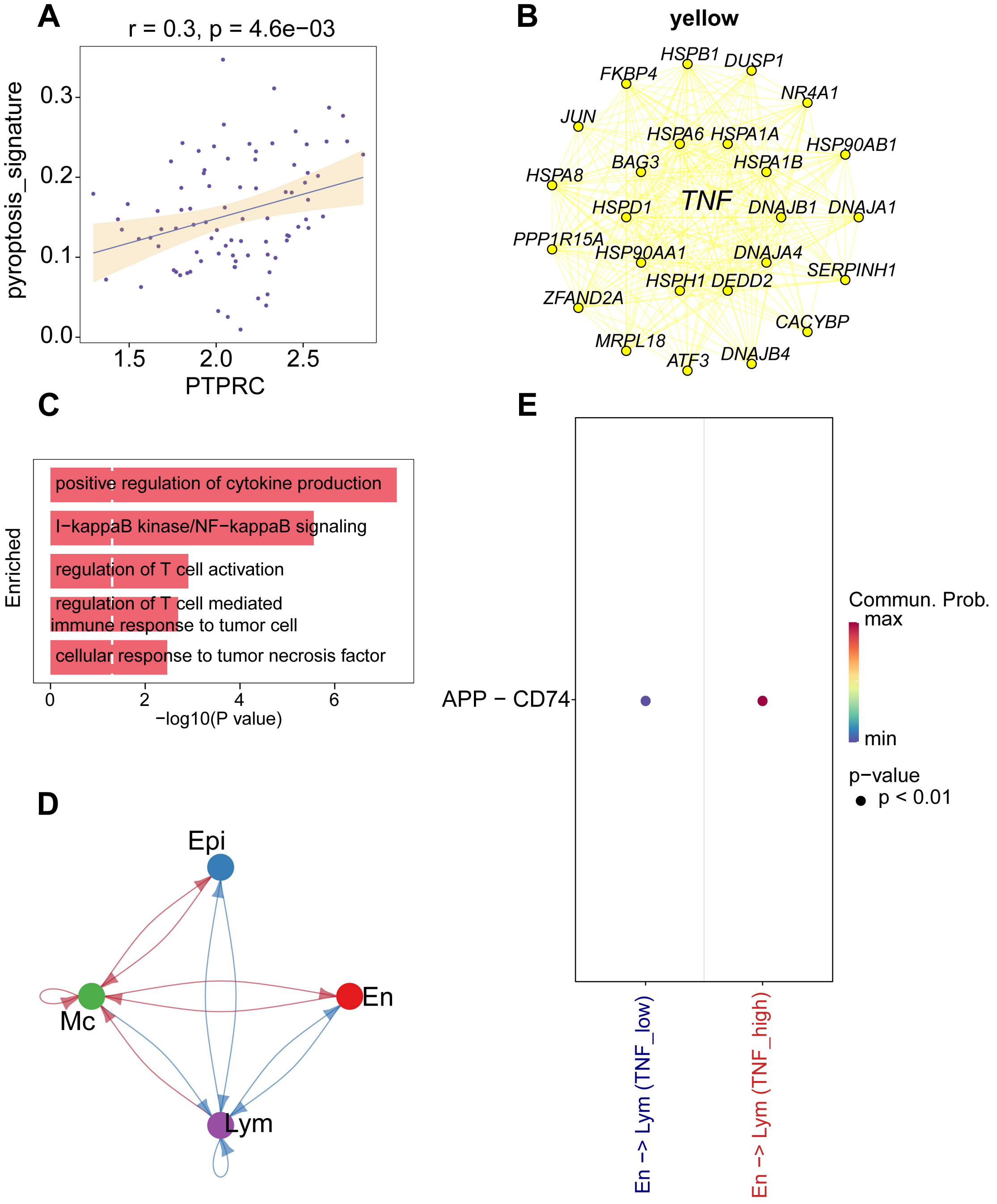

Fig. S5 (A) Correlation between the PTPRC and pyroptosis signature in UCEC.R=0.3 p < 0.05. (B) Network analysis of the correlation between *TNF* and other genes. (C)

Barplot of *BAX* module enriched function pathway; the X-axis is -log (p-value), the Y-axis is the different pathways in which genes are involved. (D) Cellchat analysis of the communication between four kind of cells. (E) Communication and ligand-receptor interaction between Endothelial cells and lymphocyte cells showing in the dotplot (high *TNF* versus low *TNF*).

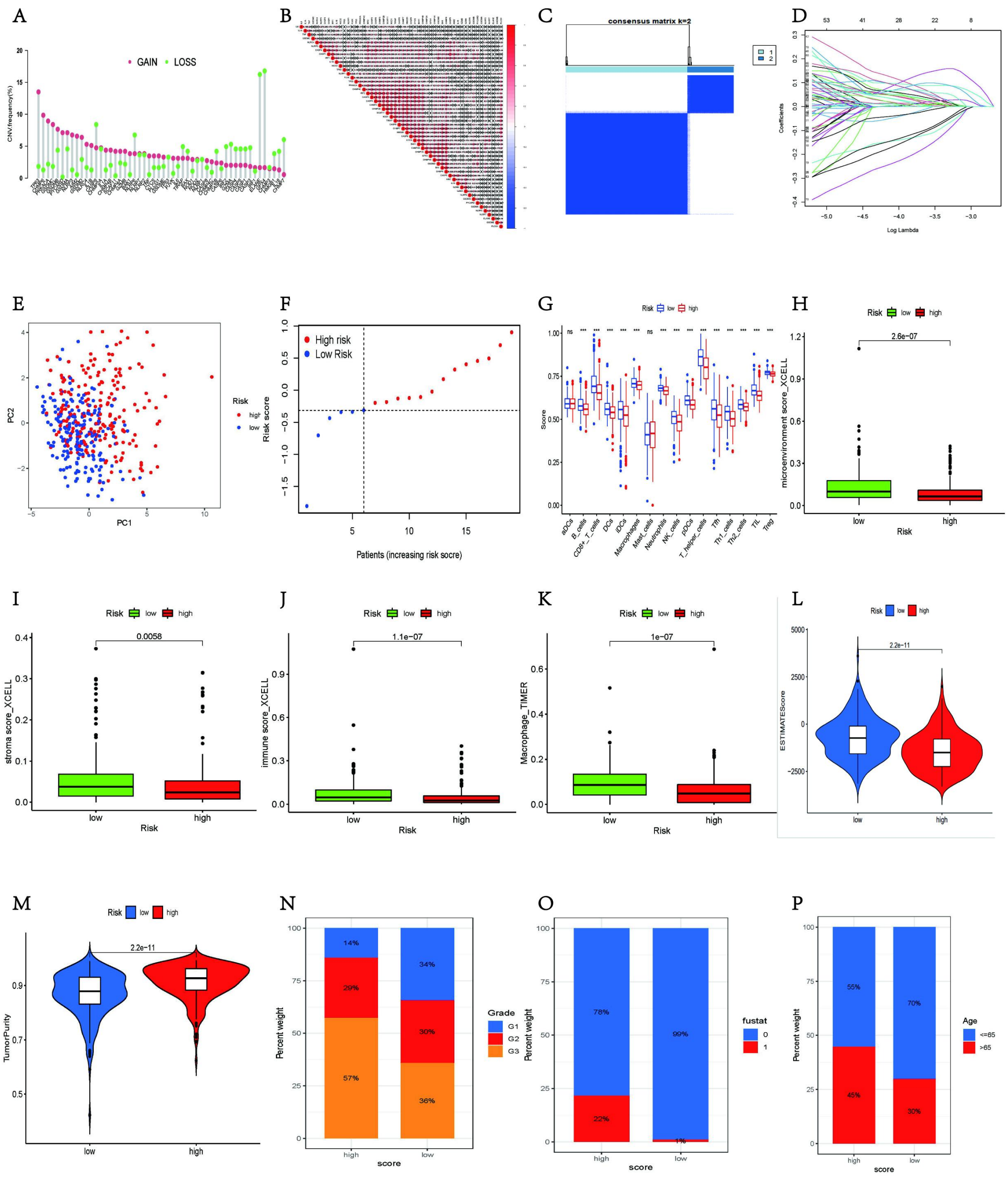

Fig. S6 (A) Gain and loss of CNV frequency. (B) The heatmap was used to visualize

these biological processes, and red represented activated pathways and blue represented inhibited pathways. The UCEC cohorts were used as sample annotations. (C) The unsupervised clustering about The difference of gene expression levels of 51 pyroptosis regulators. (D) LASSO coefficient profile plots of the prognostic related genes showing that the variations in the size of the coefficients of parameters shrink with an increasing value of the k penalty. (E) PC plot of patients in different groups. (F) The relative distribution of risk score and patient in low- or-high-risk groups. (G) Boxplots depicting the cell immune responses difference between two groups. (H-K) Boxplots depict the distribution of immune cells in the high-risk groups and low risk groups by XCELL and TIMER. (L) The relative distribution of ESTIMATE score was compared between risk score high versus low groups in UCEC cohort, respectively. (M) The relative distribution of tumor purity was compared between risk score high versus low groups in UCEC cohort, respectively. (N) The proportions of three grade groups in the low- or high- risk groups. (O) The proportions of fustat high risk and low risk. (P) The proportions of age higher 65 and low 65 groups.

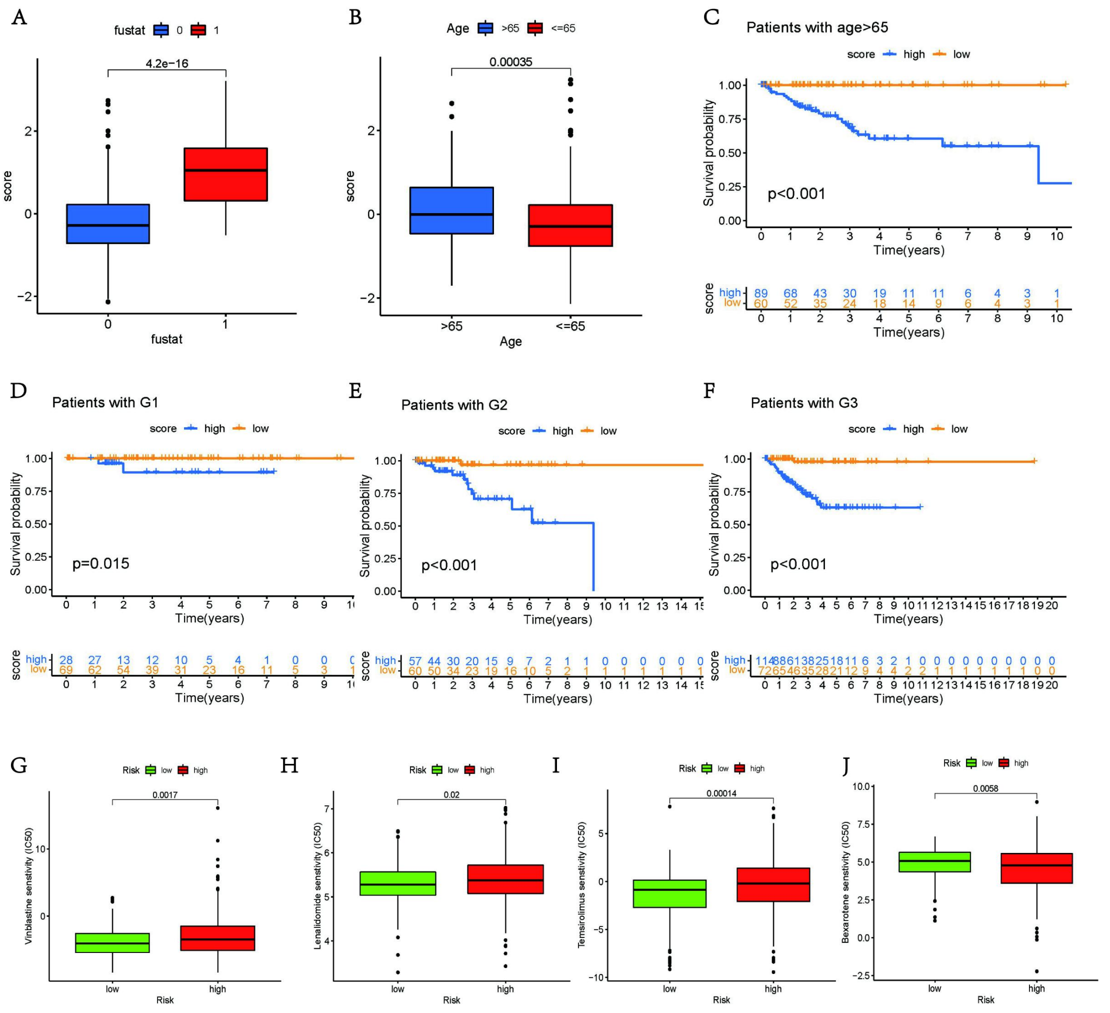

Fig. S7 (A) The difference of risk score between two fustat groups. (B) The difference of risk score between two age groups. (C-F) Kaplan - Meier curves showing progression-free survival in *GEPIA 2* in UCEC Cohort stratified according to high vs low expression of age(C),G1(D),G2(E),G3(F). (G-J) The relative distribution of drugs sensitivity were compared between risk score high versus low groups in UCEC cohort, respectively.

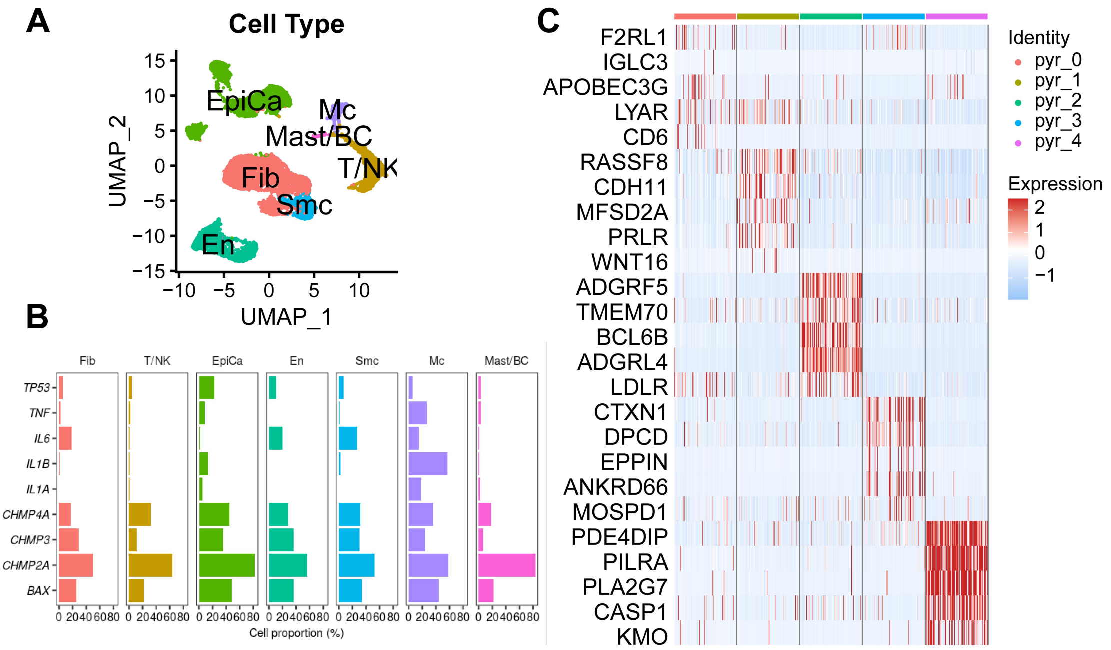

Fig. S8 (A) UMAP graph showing the expression of 7 cell types, including myeloid cells, endothelial cells, fibroblasts, smooth muscle cells, Mast/BC, T/NK, and epithelial cells. (B) Percentage of pyroptosis regulators in each cell type. (C) Heatmap of DEGs of unsupervised clustering by five clusters of pyroptosis regulators.

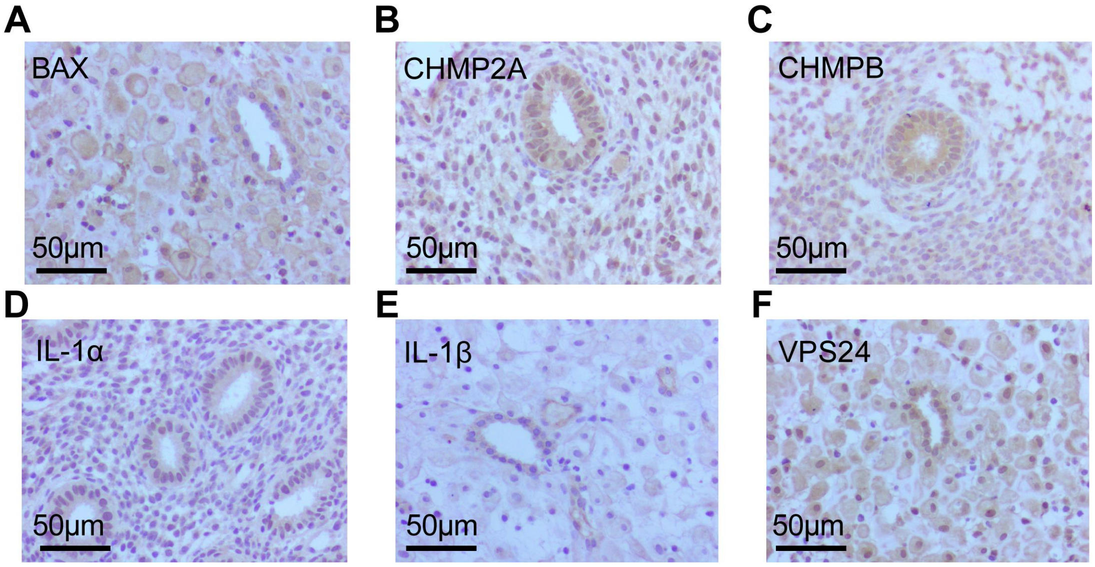

Fig. S9 (A-F) Immunohistochemical analysis was performed on normal tissues (n = 18) (Scale bars: 50.0 μm).

Table

Table S1 Comparison of basic data from eligible selected endometrial cancer patients and controls. Including age, tumor size, histology, histological grading.

Patient		Ca1	Ca2	Ca3	N1	N2	N3
Characteristic	Age	62	50	53	51	53	46
	Tumor size (cm)	2.5*2.1*1.3	1.5*1.0*0.5	1.5*1.5*0.8	-	-	-
	Histology	Adenocarcinoma	Adenocarcinoma	Adenocarcinoma	-	-	-
	Histological grading	G1	G1	G1	-	-	-

a: Patients in the experimental group were annotated as Ga1, Ca2 and Ca3, respectively. Patients in the control group were labeled as N1, N2 and N3, respectively.

b: Histological grading is divided into four levels, G1, G2, G3 and G4.

Table S2 Comparison of basic data from eligible selected endometrial cancer patients and controls. Including age, tumor size, histology, histological grading.

Patient		Ca4	Ca5	Ca6	N4	N5	N6
Characteristic	Age	43	55	63	58	63	75
	Tumor size (cm)	1.2*1.0*0.8	1.5*1.0*0.5	1.2*1.0*0.5	-	-	-
	Histology	Adenocarcinoma	Adenocarcinoma	Adenocarcinoma	-	-	-
	Histological grading	G1	G1	G1	-	-	-

a: Patients in the experimental group were annotated as Ga1, Ca2 and Ca3, respectively. Patients

in the control group were labeled as N1, N2 and N3, respectively.

b: Histological grading is divided into four levels, G1, G2, G3 and G4.

Table S3 Comparison of basic data from eligible selected endometrial cancer patients and controls. Including age, tumor size, histology, histological grading.

Patient		Ca7	C85	Ca9	N7	N8	N9
Characteristic	Age	45	71	65	57	63	58
	Tumor size (cm)	1.1*1.0*0.8	1.0*1.0*0.5	1.2*1.0*0.5	-	-	-
	Histology	Adenocarcinoma	Adenocarcinoma	Adenocarcinoma	-	-	-
	Histological grading	G1	G1	G1	-	-	-

a: Patients in the experimental group were annotated as Ga1, Ca2 and Ca3, respectively. Patients in the control group were labeled as N1, N2 and N3, respectively.

b: Histological grading is divided into four levels, G1, G2, G3 and G4.

Table S4 Comparison of basic data from eligible selected endometrial cancer patients and controls. Including age, tumor size, histology, histological grading.

Patient		Ca10	Ca11	Ca12	N10	N11	N12
Characteristic	Age	55	55	63	50	53	45
	Tumor size (cm)	1.5*1.0*0.5	1.5*1.0*0.5	1.0*1.0*0.5	-	-	-
	Histology	Adenocarcinoma	Adenocarcinoma	Adenocarcinoma	-	-	-
	Histological grading	G1	G1	G1	-	-	-

a: Patients in the experimental group were annotated as Ga1, Ca2 and Ca3, respectively. Patients in the control group were labeled as N1, N2 and N3, respectively.

b: Histological grading is divided into four levels, G1, G2, G3 and G4.

Table S5 Comparison of basic data from eligible selected endometrial cancer patients and controls. Including age, tumor size, histology, histological grading.

**Patient**		**Ca13**	**Ca14**	**Ca15**	**N13**	**N14**	**N15**
**Characteristic**	**Age**	48	55	60	55	66	49
	**Tumor size (cm)**	1.0*1.0*0.5	1.2*1.0*0.5	1.1*1.0*0.5	-	-	-
	**Histology**	Adenocarcinoma	Adenocarcinoma	Adenocarcinoma	-	-	-
	**Histological grading**	G1	G1	G1	-	-	-

a: Patients in the experimental group were annotated as Ga1, Ca2 and Ca3, respectively. Patients in the control group were labeled as N1, N2 and N3, respectively.

b: Histological grading is divided into four levels, G1, G2, G3 and G4.

Table S6 Comparison of basic data from eligible selected endometrial cancer patients and controls. Including age, tumor size, histology, histological grading.

**Patient**		**Ca16**	**Ca17**	**Ca18**	**N16**	**N17**	**N18**
**Characteristic**	**Age**	46	43	44	68	53	55
	**Tumor size (cm)**	1.2*1.0*0.8	1.5*1.0*0.5	1.2*1.0*0.5	-	-	-
	**Histology**	Adenocarcinoma	Adenocarcinoma	Adenocarcinoma	-	-	-

**Histological grading**	G1	G1	G1	-	-	-

a: Patients in the experimental group were annotated as Ga1, Ca2 and Ca3, respectively. Patients in the control group were labeled as N1, N2 and N3, respectively.

b: Histological grading is divided into four levels, G1, G2, G3 and G4.

Table S7 The primers used for RT-qPCR were as follows:

Gene	Primer nucleotide sequence (5′ to 3′)	
GAPDH	F:GCTCTCTGCTCCTCCTGTTC	R:ACGACCAAATCCGTTGACTC
BAX	F:CATGGAGCTGCAGAGGATGA	R:CTGATCAGTTCCGGCACCTT
CHMP4A	F:GCCTACCAGGACATGGACATT	R:CCATAGGCCGAGAAATGGCA
CHMPB	F:AGAGTTTGACGAGGATGAGC	R:CGGGTTTTGATGGTAGGGCT
IL1A	F:GCGTTTGAGTCAGCAAAGAAGT	R:GCCGTGAGTTTCCCAGAAGA
IL1B	F:AGCCATGGCAGAAGTACCTG	R:CCTGGAAGGAGCACTTCATCT
VSP24	F:TGCAGAGGAGATTTCAACAGC	R:TGTTTCGGGTCCACTGATTTC
